# Multispecific Antibody Development Platform Based on Human Heavy Chain Antibodies

**DOI:** 10.3389/fimmu.2018.03037

**Published:** 2019-01-07

**Authors:** Starlynn C. Clarke, Biao Ma, Nathan D. Trinklein, Ute Schellenberger, Michael J. Osborn, Laure-Hélène Ouisse, Andrew Boudreau, Laura M. Davison, Katherine E. Harris, Harshad S. Ugamraj, Aarti Balasubramani, Kevin H. Dang, Brett Jorgensen, Heather Anne N. Ogana, Duy T. Pham, Payal P. Pratap, Preethi Sankaran, Ignacio Anegon, Wim C. van Schooten, Marianne Brüggemann, Roland Buelow, Shelley Force Aldred

**Affiliations:** ^1^Teneobio, Inc., Menlo Park, CA, United States; ^2^Centre de Recherche en Transplantation et Immunologie, Inserm UMR 1064, Université de Nantes, Nantes, France

**Keywords:** heavy chain antibodies, VH domains, domain antibodies, antibody discovery platform, next-generation sequencing, repertoire sequencing, transgenic rats, B cell development

## Abstract

Heavy chain-only antibodies (HCAbs) do not associate with light chains and their V_H_ regions are functional as single domains, forming the smallest active antibody fragment. These V_H_ regions are ideal building blocks for a variety of antibody-based biologics because they tolerate fusion to other molecules and may also be attached in series to construct multispecific antibodies without the need for protein engineering to ensure proper heavy and light chain pairing. Production of human HCAbs has been impeded by the fact that natural human V_H_ regions require light chain association and display poor biophysical characteristics when expressed in the absence of light chains. Here, we present an innovative platform for the rapid development of diverse sets of human HCAbs that have been selected *in vivo*. Our unique approach combines antibody repertoire analysis with immunization of transgenic rats, called UniRats, that produce chimeric HCAbs with fully human V_H_ domains in response to an antigen challenge. UniRats express HCAbs from large transgenic loci representing the entire productive human heavy chain V(D)J repertoire, mount robust immune responses to a wide array of antigens, exhibit diverse V gene usage and generate large panels of stable, high affinity, antigen-specific molecules.

## Introduction

Heavy chain only antibodies (HCAbs) occur naturally in camelids (V_H_H) and cartilaginous fish (V_NAR_), where they form a functional paratope using only the heavy chain variable domain without light chain pairing (1–4). Since the initial discovery of V_H_Hs in 1993, extensive research has demonstrated that HCAbs have both therapeutic and developability characteristics equivalent to conventional antibodies (5–8). Furthermore, HCAbs are smaller than standard Ig molecules since they lack light chains, which may facilitate targeting of epitopes not readily accessible to classic antibodies, including narrow protein clefts and enzyme active sites (1, 9, 10). In addition, the V_H_ regions of HCAbs form stable polypeptides in the absence of an Fc and can therefore be expressed as single domain proteins. As the smallest functional antibody fragment, these proteins may be ideal for certain applications that benefit from improved tissue penetrance, central nervous system accessibility and amenability to alternative routes of administration. V_H_ domains from HCAbs are also attractive building blocks for multispecific biologics, facilitating easy combination of different antigen-specific variable regions within a single molecule (10–14).

The utility of HCAbs has spurred the development of multiple antibody discovery platforms based on V_H_H or V_NAR_ sequences (15–19). However, despite some promising clinical results with camelid-derived HCAbs, there remain significant drawbacks to developing these molecules as drugs. Of principle concern is the potential immunogenicity of non-human sequences, which requires humanization of framework regions prior to clinical development. This process prolongs the drug discovery pipeline and can potentially impact important biophysical characteristics including stability and antigen affinity (20, 21). In addition, practical considerations regarding the care and immunization of large animals, such as llamas, limit accessibility to *in vivo* matured molecules and reduce the number of individuals that can be immunized with a given antigen. Antibody repertoire sequencing has revealed that individuals in the same immunization cohort produce widely variable antibody repertoires, therefore, including multiple animals in every immunization campaign ensures maximum sequence diversity and improves selection of optimal therapeutic candidates (22).

Several groups have initiated efforts to produce fully human HCAbs *in vitro*, however, many of these attempts have been stymied by the difficulty of preventing aggregation due to exposure of hydrophobic heavy chain residues normally shielded by light chain binding (23–26). Encouragingly, a few groups have reported success in engineering transgenic mice to produce human HCAbs, but limited information has been publicly available regarding whether conventional human (V_H_DJ_H_) sequences can be efficiently expressed as HCAbs in transgenic rodents to generate diverse and high affinity molecules (17, 27).

We have created an antibody discovery platform that addresses these challenges through antibody repertoire sequencing in transgenic rats, called UniRats, that produce heavy chain only antibodies with fully human variable domains, termed UniAbs™. UniRats express UniAbs due to genomic insertion of large transgenic loci accommodating the full repertoire of functional human V_H_, D, and J_H_ genes, while endogenous rat Ig expression has been silenced by targeted disruption of the IgH, Igκ and Igλ loci with inserted zinc-finger-nuclease constructs (22, 28–30). Our discovery approach combines next-generation sequencing (NGS) of the antibody repertoires produced by immunized UniRats with high-throughput gene assembly and expression, meaning that hundreds of unique antibodies are analyzed for each antigen and large sets of diverse, target-specific candidates are rapidly identified (22).

In this report we describe construction of UniRat strains that produce distinct human HCAb repertoires following immunization with a wide range of antigens. We find that B cell development within these animals is extensive and near normal numbers of B cells are observed in the bone marrow and peripheral lymphoid organs, despite a lack of IgM production. We demonstrate that UniAbs are stable proteins similar to light chain-containing antibodies in terms of target affinity, yield, stability, and aggregation propensity. Bioinformatics analysis of a large set of UniAbs revealed that hydrophilic residues are present at a higher frequency in UniAb complementarity determining regions (CDRs) compared to antibodies utilizing light chains, possibly as an adaptation to the absence of V_L_ domains. Furthermore, we crystalized a UniAb V_H_ and found that while its structure is similar to conventional human heavy-chain domains, an engineered framework mutation and mutations occurring during *in vivo* maturation reduce the surface hydrophobicity of this molecule compared to typical human V_H_s. By combining genetic engineering with natural differentiation by the immune system, we show that diverse V_H_DJ_H_ repertoires of high affinity fully human HCAbs can be rapidly generated, expressed, and characterized, potentially serving as building blocks to accelerate multispecific human antibody development.

## Results

### Construction of UniRat Strains

**Figure 1:**
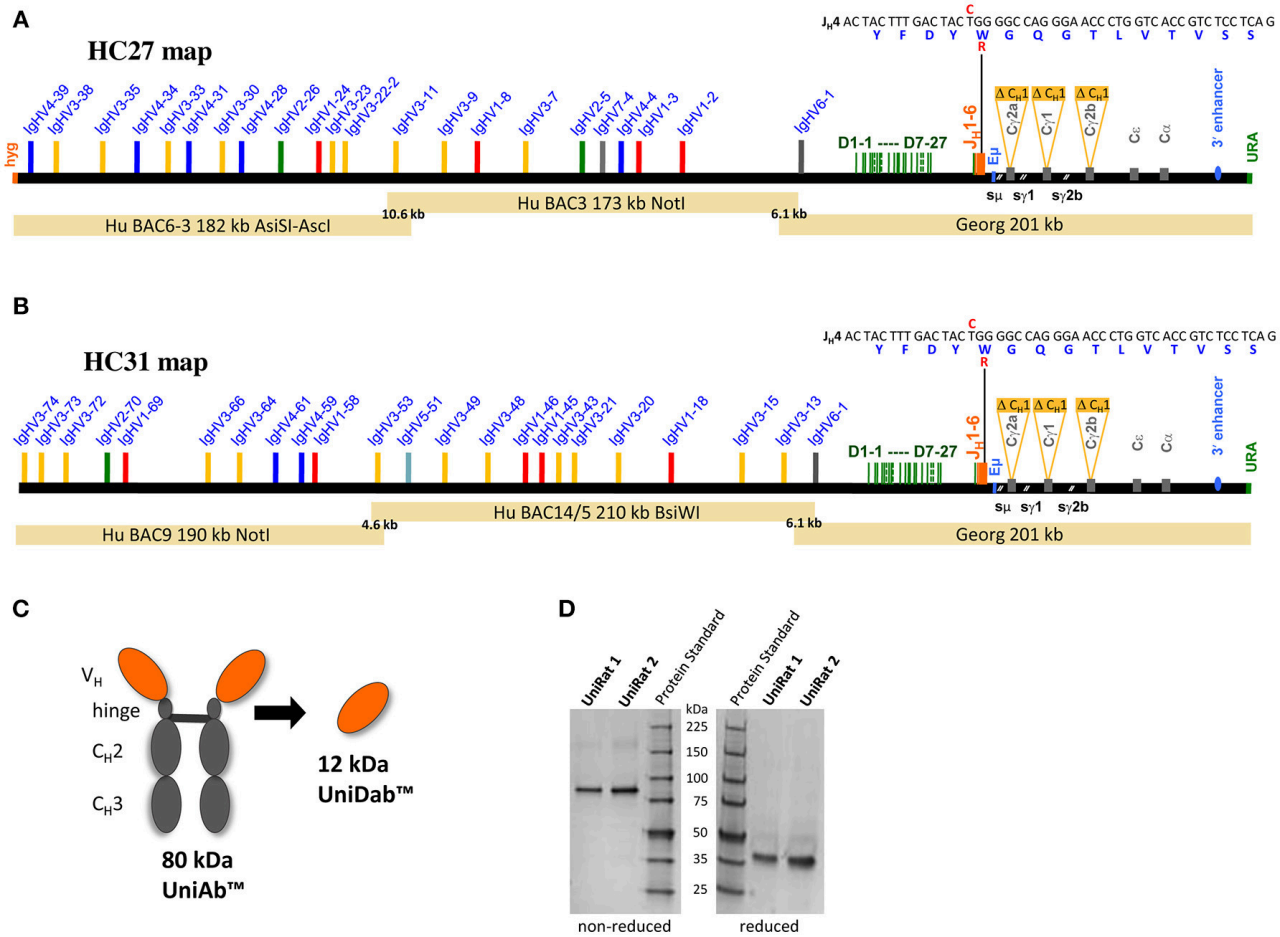
Construction and validation of UniRat transgenic animals. Bacterial artificial chromosomes (BACs) were used for microinjection into fertilized oocytes from triple Ig KO rats, resulting in the creation of two UniRat strains expressing different sets of human V genes, HC27 **(A)** and HC31 **(B)**. UniAbs lack C_H_1 domains and light chains, resulting in a molecular weight of approximately 80 kDa. Heavy chain V_H_s from UniRats may also be converted to single domain antibodies (UniDabs^TM^) **(C)**. SDS-PAGE analysis of UniRat IgG purified from the serum of two different animals confirmed that the predominant species runs at ~80 kDa under non-reducing conditions and 40 kDa after reduction of cysteine bonds, consistent with the expected molecular weight of UniAbs **(D)**. Native antibodies are not expressed by UniRats as their endogenous Ig loci have been silenced.

Given the large size of the full human V_H_ repertoire, two separate UniRat strains were generated, (termed HC27 and HC31), expressing different parts of a complete functional human V gene repertoire together with the full suite of human D and J_H_ genes (Figures 1A,B). These strains were created through DNA microinjection of overlapping BACs (bacterial artificial chromosomes) into fertilized rat oocytes. In addition, the animals were bred to homozygosity to acquire a triple Ig knockout background, where the native rat IgH, Igκ, and Igλ loci were inactivated (22, 28–30). Large IgH loci on BACs carrying human V_H_, D and J_H_ segments in germline configuration linked to rat Cγ genes were compiled as detailed previously (29, 31), except that a new assembly of rat C_H_ genes lacking C_H_1 domains was used. Each chimeric IgH locus contained multiple BACs with short 5′ and/or 3′ overlaps (Figures 1A,B), which promoted seamless tandem integration. This resulted in genomic co-integration and reconstitution of operational IgH loci, which, after DNA rearrangements, allowed production of UniAbs comprised of two covalently linked heavy chains. Germline sequences were used for all human V_H_, D and J_H_ genes with one exception; the J4 gene sequence encodes the mutation W101R [Kabat numbering scheme (32)]. This mutation has been shown to reduce antibody hydrophobicity and thus it was included to compare biophysical characteristics of UniAbs bearing the arginine variant compared to the wild type tryptophan (33).

After successful genomic co-integration, founders were identified through qPCR analysis and bred to homozygosity. Analysis of serum Ig from two separate UniRats confirmed production of a single antibody species running at approximately 80 kDa under non-reducing and 40 kDa under reducing conditions on a protein gel, consistent with the expected molecular weight of UniAbs (Figures 1C,D).

**Figure 2:**
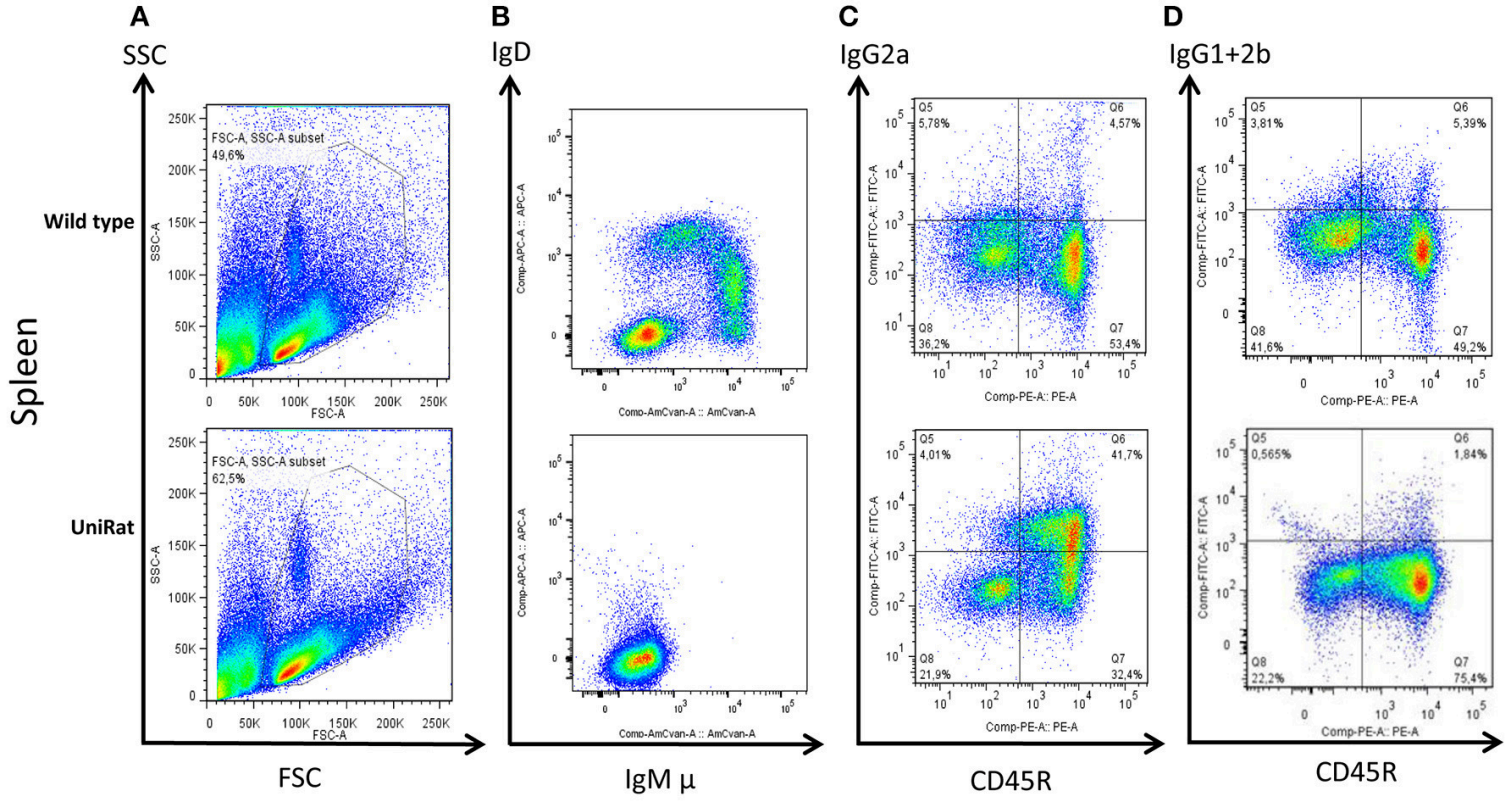
UniRats exhibit normal B cell development. Flow cytometry analysis of B cells isolated from the spleens of wild type rats (*n* = 4, Sprague Dawley background) and UniRats (*n* = 4, strain HC27), demonstrating robust production of lymphocytes and extensive B cell development. Gating of lymphocytes by forward and side scatter **(A)**, IgM and IgD are not expressed by UniRats due to a lack of Cμ and Cδ sequences **(B)**. UniRats express higher levels of IgG2a^+^ B cells compared to wild type animals **(C)**, but slightly lower levels of IgG1^+^ and IgG2b^+^ B cells **(D)** (gated on T cell receptor (TCR) negative, viable lymphocytes). Representative dot plots are shown.

Following creation of the UniRat strains, B cell development was analyzed by flow cytometry. Four wild type rats and four UniRats (strain HC27) were immunized for 21-days with β-galactosidase. Subsequently, spleens, lymph nodes and bone marrow were isolated from these animals and the cells were dissociated and analyzed by flow cytometry. IgM^+^ and IgD^+^ B cells are not produced by UniRats, however extensive B cell development occurs as evidenced by the production of Ig and comparable levels of B cells in secondary lymphoid organs, (Figure 2, Supplemental Figure 1, Table 1), in agreement with previous work showing that IgM is not required for pre-B cell signaling (17). UniRats express high levels of IgG2a^+^ B cells, while IgG1^+^ and IgG2b^+^ B cells are produced at comparable levels to wild type rats (Figures 2C,D, Supplemental Figures 1D,E).

**Table 1:** UniRats contain high levels of lymphocytes within their primary and secondary lymphatic organs.

	**Spleen**				**Bone marrow**				**Lymph node**			
	**T cells**	**B cells**	**IgG2a^**+**^ B cells**	**IgG1/2b^**+**^ B cells**	**T cells**	**B cells**	**IgG2a^**+**^ B cells**	**IgG1/2b^**+**^ B cells**	**T cells**	**B cells**	**IgG2a^**+**^ B cells**	**IgG1/2b^**+**^ B cells**
**WT** **(*n =* 4)**	67.4 (36)	56.6 (20.7)	8.5 (6.4)	5.2 (1.7)	25.8 (0.5)	10.8 (2.7)	0.4 (0.1)	0.2 (0.04)	7 (3.3)	3.5 (2)	0.3 (0.2)	0.2 (0.1)
**HC27** **(*n =* 4)**	127 (46.4)	166 (49.2)	112 (41.2)	4.3 (2.8)	1.4 (0.6)	11 (4.7)	2.5 (1.7)	0.3 (0.1)	12.1 (4.2)	8.6 (6.2)	4.7 (3.3)	0.2 (0.1)

*Lymphocytes were isolated from the spleens, bone marrow, and lymph nodes of wild type rats and UniRats that had been immunized with β-galactosidase. The cells were then gated on viable cells, followed by staining for TCR, CD45R, IgG2a, and IgG1/2b expression. Average values in millions are reported for four animals, with standard deviation indicated in parenthesis. UniRats contained comparable or slightly elevated numbers of total T and B cells as compared to wild type rats, apart from the bone marrow where fewer T cells were observed. A larger number of the UniRat B cells express IgG2a as compared with wild type rats. Representative flow plots of lymphocytes isolated from spleens, bone marrow, and lymph nodes of HC27 and wild type rats are shown in Supplemental Figure 1*.

### High-throughput UniAb Discovery Enabled by Antibody Repertoire Sequencing

**Figure 3:**
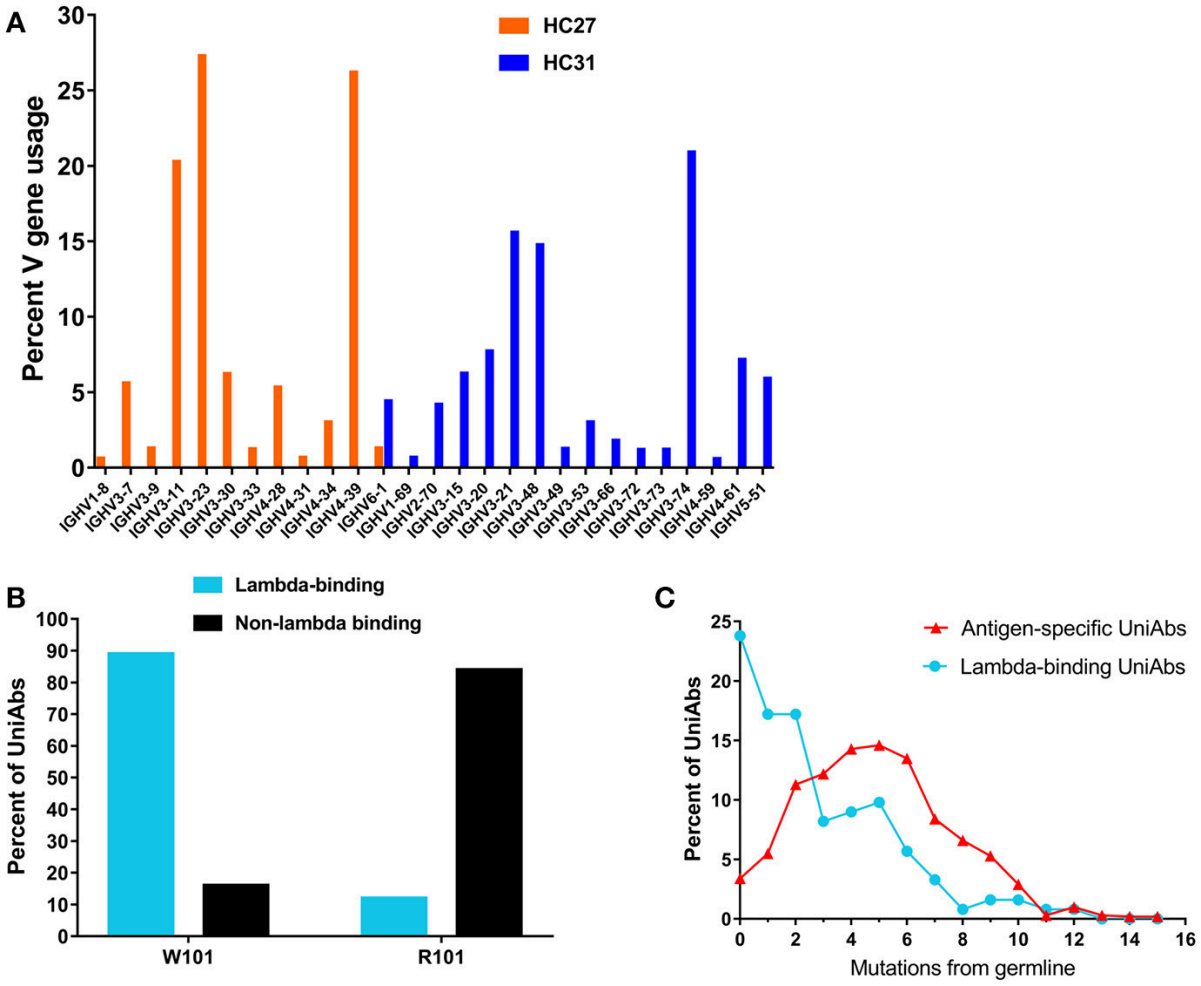
Characterization of UniRat V gene usage, light chain association, and somatic hypermutation. V gene usage was determined by next-generation sequencing (NGS) of total Ig RNA from 55 HC31 UniRats and 79 HC27 UniRats immunized with one of 20 different antigens. Each column reflects usage frequency for a single V gene averaged from UniRats of either the HC27 or HC31 strains **(A)**. Approximately 50–70% of the V genes encoded by the UniRat recombinant Ig loci were detectable at more than 0.35%, the limit of detection. A total of 2,426 UniAbs from nine immunization campaigns (Table 2) were assessed by ELISA for association with recombinant human lambda light chain protein. 122 UniAbs bound to lambda protein, of which 89% contained the wild type tryptophan at position 101 in framework 4. In contrast, 84% of sequences that did not bind lambda light chain contained the introduced framework 4 mutation W101R, suggesting a strong relationship between this mutation and lambda association **(B)**. UniAbs from the immunizations listed in Table 2 were categorized as either antigen-specific or lambda-binding and the members of each group were then binned according to somatic hypermutation (SHM) in their heavy chain variable regions (x-axis). The number of UniAbs in each bin was converted to a percent (y-axis) and graphed, revealing that antigen-specific UniAbs generally display higher levels of SHM compared to UniAbs that associate with lambda light chain **(C)**.

The functional human V_H_ repertoire consists of approximately 44 genes from seven V_H_ families, all of which are present in either the HC27 or HC31 UniRat strain (34). To analyze V gene usage in these animals, 134 UniRats (55 HC31 and 79 HC27) were each immunized with one of 20 different antigens and NGS was used to profile all expressed Ig transcripts in RNA isolated from lymph node B cells. The HC27 and HC31 strains contain 22 and 23 different V genes, respectively, and 50–70% of these V_H_s were detected at frequencies above the limit of detection (0.35%) (Figure 3A). Animals of both strains showed bias toward select V genes from either the IgHV3 or IgHV4 families and used these V_H_s with increased frequency. Consistent with our results, natural human antibody repertoires also show unequal V gene usage, and in particular a preference for IgHV3 has been noted (35, 36).

Validated UniRat strains were tested for functionality by undertaking immunization and sequence-based discovery campaigns with a panel of 10 recombinant protein antigens, similar to the approach outlined in Harris et al. (22) (Table 2). Briefly, after immunization with recombinant proteins and standard adjuvants, antigen-specific serum titers were measured by ELISA and found to be positive for all campaigns. Subsequently, lymphocytes from draining lymph nodes were collected and RNA from these cells was isolated. Generally, between 6 and 12 individuals representing the two UniRat strains were included in each immunization campaign to expand the overall antibody repertoire diversity. Following NGS-enabled sequencing of total Ig RNA, quantitative analysis of the heavy-chain repertoire supported identification of the most prevalent V_H_ sequences and guided high-throughput gene assembly of several hundred UniAb candidates for each immunized antigen. In total, 2,600 candidate UniAbs were expressed by transfection of gene-assembled cDNA constructs into HEK293 cells, and each supernatant containing secreted antibody was assayed to determine antigen specificity by ELISA.

**Table 2:** UniRats efficiently produce antigen-specific UniAbs in response to antigen challenge.

**Antigen**	**Antigen-specific/Total UniAbs screened**	**Antigen specific (%)**	**Total unique clonotype families screened**	**Antigen-specific CDR3 sequence families**	**Lambda binding sequences (%)**
Human BCMA	54/309	17	171	28	4.9
Human and cynomolgus CD38	42/173	24	96	37	1.2
Human PSMA	41/265	15	97	15	0.8
Human and cynomolgus PD-1	39/252	15	85	20	2.4
Human and cynomolgus BCMA	120/334	36	111	28	9
Mouse CD38	91/218	42	89	37	7.8
Human and cynomolgus CD22	55/277	20	123	22	2.5
Human PD-L1	61/329	19	189	37	5.8
HIV-1 gp120 envelope protein	27/174	16	100	14	Not tested
Human PSCA	30/269	11	70	10	8.9

*Ten different immunization campaigns using both human and animal proteins were carried out. On average 260 unique UniAbs representing 113 different clonotype sequence families were analyzed for each campaign. Candidates were screened for antigen specificity as well as binding to recombinant human lambda light chain by ELISA. On average, 21.5% of all candidate UniAbs were antigen positive, while only 5% of UniAbs showed association with lambda light chain*.

This sequence-directed gene assembly approach ensured that each assayed V_H_ was unique, resulting in a primary screening set for each antigen composed of multiple members from approximately 100 distinct clonotype sequence families (113 on average), as defined by CDR3 similarity of at least 80% (Table 2). The percent of candidates showing antigen-specific binding by ELISA varied by target, ranging from 11 to 42% of assayed UniAbs, representing an average of 25 unique clonotype sequence families per antigen (Table 2). In addition, primary screen leads were routinely found to have single digit nM equilibrium dissociation constants (K_D_), as is commonly observed for discovery approaches based on conventional antibodies (Table 3, Supplemental Figure 2).

**Table 3:** UniRats produce high-affinity UniAbs in response to antigen challenge.

**Antigen**	**K_**D**_ of highest affinity lead (nM)**
Mouse CD38	0.2
Human BCMA	0.3
Cynomolgus CD38	1.0
Human CD38	2.6
Human PD-L1	3.6
Human PSMA	7.1
Cynomolgus BCMA	8.3

*Affinities to recombinant protein antigens were determined using biolayer interferometry (Octet) for UniAbs from several immunization campaigns. Single digit nanomolar affinities were observed for UniAbs targeting a variety of antigens. Sensorgrams are included in Supplemental Figure 2*.

Natural human antibodies are expressed as V_H_-V_L_ heterodimers, thus binding to light chain proteins is a potential liability for human V_H_s that are produced as HCAbs. A tendency to bind light chain may also be correlated with poor colloidal stability, as it indicates possible exposure of hydrophobic residues in the former light-chain binding interface (33). Therefore, we investigated whether UniAbs associate with recombinant human lambda light chain by ELISA. The frequency of lambda binders isolated from each immunization campaign was found to vary from 0.8 to 9%, with 5% of all UniAbs showing this behavior (Table 2). After analyzing UniAbs that associate with lambda light chain for sequence commonalities, we found that nearly 90% of these UniAbs displayed a tryptophan at position 101, whereas the majority (84%) of non-lambda reactive sequences exhibited the arginine variant engineered into the J_H_4 gene at this residue (Figure 3B). While it is possible that another feature of the J_H_4 sequence contributed to reduced association with lambda, no association between either J_H_3 or J_H_5 usage and reduced lambda binding was observed despite the high degree of sequence similarity among IgHJ4^*^01, IgHJ3^*^01, and IgHJ5^*^01 (34). Interestingly, we note that additional mutations within a V_H_ region can impact the tendency for lambda association, as a tryptophan vs. arginine at position 101 was not entirely predictive of lambda association. For example, an in-depth sequence analysis of 11 UniAbs from one CDR3 sequence family in which some members bound to lambda while others did not, revealed that mutations within the CDR3 loop abrogated lambda association, even when a tryptophan was present at position 101 (Supplemental Figure 3). However, analysis of the complete UniAb data set did not identify any specific mutations correlated with light chain binding in general, apart from W101R, suggesting that other mutations affecting lambda association may be unique to each clonotype.

Analysis of somatic hypermutation (SHM) revealed that UniAbs that associate with lambda protein have fewer mutations from germline compared to UniAbs which do not bind lambda, with approximately 25% being completely germline (Figure 3C). In contrast, <5% of antigen-specific, non-lambda reactive UniAbs had germline sequences, with the majority bearing two or more mutations, consistent with analysis of antibodies composed of two heavy and two light chains (H2L2) produced after immunizations with comparable protocols (22) (Figure 3C).

### UniAbs Are Thermostable

**Figure 4:**
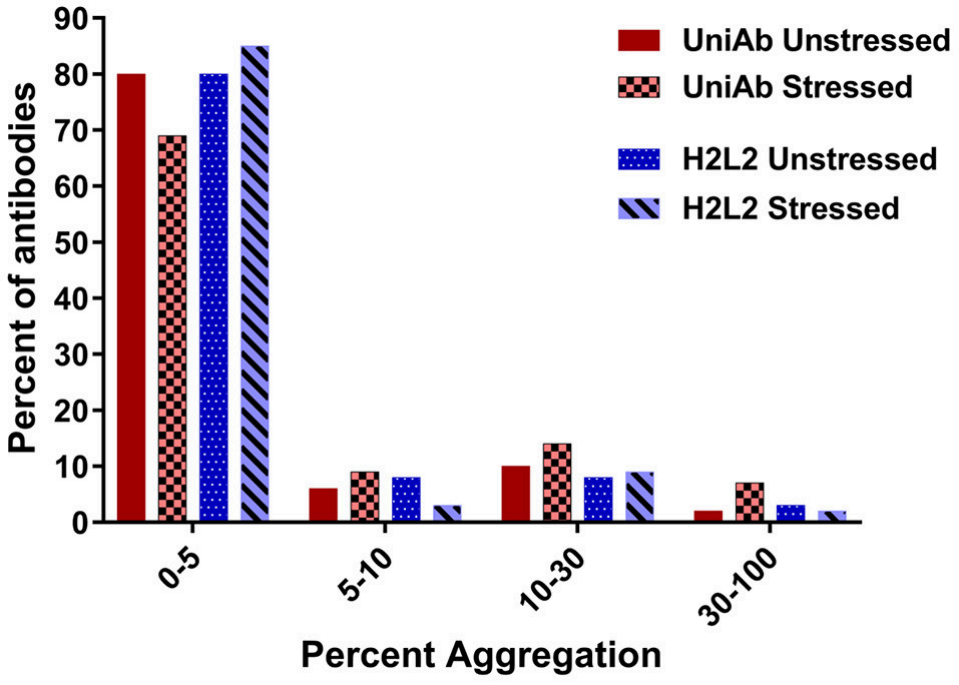
UniAbs are thermostable.To compare UniAbs to H2L2 antibodies, 124 unique UniAb sequences (a subset of the larger collection listed in Table 2) were expressed and purified side-by-side with 86 different H2L2 antibodies from three immunization campaigns that used a similar discovery workflow but were derived from transgenic rats expressing human heavy chains containing a C_H_1 domain as well as a fixed human light chain^22^. Compared to H2L2 antibodies, UniAbs exhibit similar aggregation propensities before and slightly increased aggregation after temperature stress (1 week at 37°C) as measured by size exclusion chromatography (SEC).

To investigate the inherent aggregation propensity of UniRat-derived heavy chain only antibodies, a subset of 124 UniAb sequences were purified and analyzed. The test set was composed of approximately 30% antigen specific UniAbs and 70% randomly selected, non-antigen binding molecules from the above listed immunization campaigns (Table 2). A comparison set of 86 light chain-containing antibodies was constructed from immunizations targeting human CD3, human kappa light chain and human serum albumin that had been derived from transgenic rats expressing human antibodies containing fixed light chains [animals described in Harris et al. (22)]. All antibodies were expressed, purified and evaluated for protein expression, then percent aggregation was determined by size exclusion chromatography (SEC-HPLC). On average, UniAbs expressed at 1 mg/mL, slightly higher than the comparison set of H2L2 antibodies (0.8 mg/mL) and exhibited a similar aggregation propensity prior to temperature stress (Figure 4 and data not shown). Following temperature stress (1 week at 37°C), UniAbs had somewhat reduced colloidal stability compared to H2L2 antibodies. However, the majority of antibodies, 69% of UniAbs and 85% of H2L2 antibodies, displayed < 5% aggregate (Figure 4). To further investigate thermostability, a subset of six different UniAbs targeting human BCMA were analyzed by differential scanning calorimetry (DSC). The T_m_ values of the V_H_ domains ranged from 59.1° to 66.8°C, within the range expected for conventional H2L2 V_H_ domains (Supplemental Table 1) (37, 38).

### Analysis of Amino Acid Usage in UniAb CDRs

We hypothesized that the absence of light chains could impose unique *in vivo* selection pressure on UniAbs, possibly leading to changes in amino acid usage compared to light chain containing antibodies. To investigate this question, we analyzed large collections of both UniAbs and H2L2 antibodies categorized by V gene to control for variation in amino acid frequency between germline V gene sequences. To maximize the size of our UniAb test set we focused our analysis on two frequently used V-genes, IgHV3-23 and IgHV4-39, and collated all sequences that had been expressed from 30 separate immunization campaigns, totaling 4,275 and 3,296 IgHV3-23 and IgHV4-39 UniAbs, respectively. We applied a similar strategy to build a comparison set of H2L2 fixed-light chain antibodies, totaling 2,617 IgHV3-23 and 1,598 IgHV4-39 heavy-chain sequences compiled from 32 distinct immunizations. We reasoned that fixed light chain H2L2 antibodies were an ideal comparison set because they were derived from animals of similar genetic backgrounds using comparable immunization and NGS protocols and selection algorithms (22). To facilitate identification of broad trends, we assigned all amino acids to one of four categories: hydrophobic, charged, polar uncharged, and special cases and then determined the frequency of each category within the CDR1, 2, and 3 loops of each UniAb and H2L2 antibody set (Table 4 and Supplemental Table 2). Next, we quantified the percent change in each category between the H2L2 and UniAb groups by subtracting each H2L2 frequency from the corresponding UniAb frequency and converting the decimal value to a percent (Table 4). Interestingly, we found that charged and polar uncharged residues were present with increased frequency in the CDR3 regions of both the IgHV3-23 and IgHV4-39 UniAb test sets, while hydrophobic amino acid usage was decreased compared to the H2L2 antibodies. A Mann-Whitney statistical test confirmed that these differences were significant. Similar, although less striking, trends in amino acid usage were also observed within the CDR1 and CDR2 loops.

**Table 4:** Amino acid usage varies between UniAbs and H2L2 antibodies.

	**V3-23**		**V4-39**	
**CDR_category**	**% Difference: UniRat vs. H2L2**	***p*-value**	**% Difference: UniRat vs. H2L2**	***p*-value**
CDR1: hydrophobic	−0.2		−0.7
CDR1: special cases	−0.3		0.0
CDR1: charged	−1.1		−0.4
CDR1: polar uncharged	+1.6		+1.1
CDR2: hydrophobic	−1.5		−1.9
CDR2: special cases	−0.6		−0.3
CDR2: charged	+0.4		+2.4	1.0E−24
CDR2: polar uncharged	+1.7		−0.2
CDR3: hydrophobic	−8.7	1.3E−223	−7.4	1.4E−101
CDR3: special cases	+0.1		−1.9
CDR3: charged	+5.0	2.7E−87	+3.4	1.6E−29
CDR3: polar uncharged	+3.6	1.4E−44	+5.8	1.2E−65
**Categories**				**Amino Acids**
Hydrophobic				A, V, I, L, M, F, Y, W
Special cases				C, G, P
Charged				R, H, K, D, E
Polar uncharged				S, T, N, Q

*Amino acids were categorized into one of four classes as indicated. The frequency of each category in the CDR1, CDR2, and CDR3 was determined for a large set of UniAbs and fixed-light chain H2L2 antibodies that use either IGHV3-23 or IGHV4-39. The differences in frequencies for each category were then compared between UniAbs and H2L2 antibodies, revealing that UniAbs contain more charged and polar uncharged amino acids and fewer hydrophobic residues. These trends were particularly notable in the CDR3 region. Differences of more than 2% are highlighted in the table below and were found to be statistically significant using a Mann-Whitney test. Usage frequencies and standard deviations are included in Supplemental Table 2*.

### Crystal Structure of a UniAb V_H_

To provide further insight into UniAb V_H_ architecture, we crystallized a complex of a UniAb V_H_ (IgHV3-23) bound to its antigen, the extracellular domain of human BCMA. Purified complexes were isolated by SEC and crystals were generated using sitting drop vapor diffusion in a PEG ion solution. Diffraction data was collected at the Australian synchrotron and molecular replacement and refinement was used to solve the structure to a resolution of 2.6 Å (Supplemental Table 3). A crystal structure of a single domain antibody [Protein Data Bank (PDB) 3ZHK] and a structure of BCMA (PDB 4ZFO chain F) were used as molecular replacement models for the UniAb V_H_ and BCMA chains, respectively.

**Figure 5:**
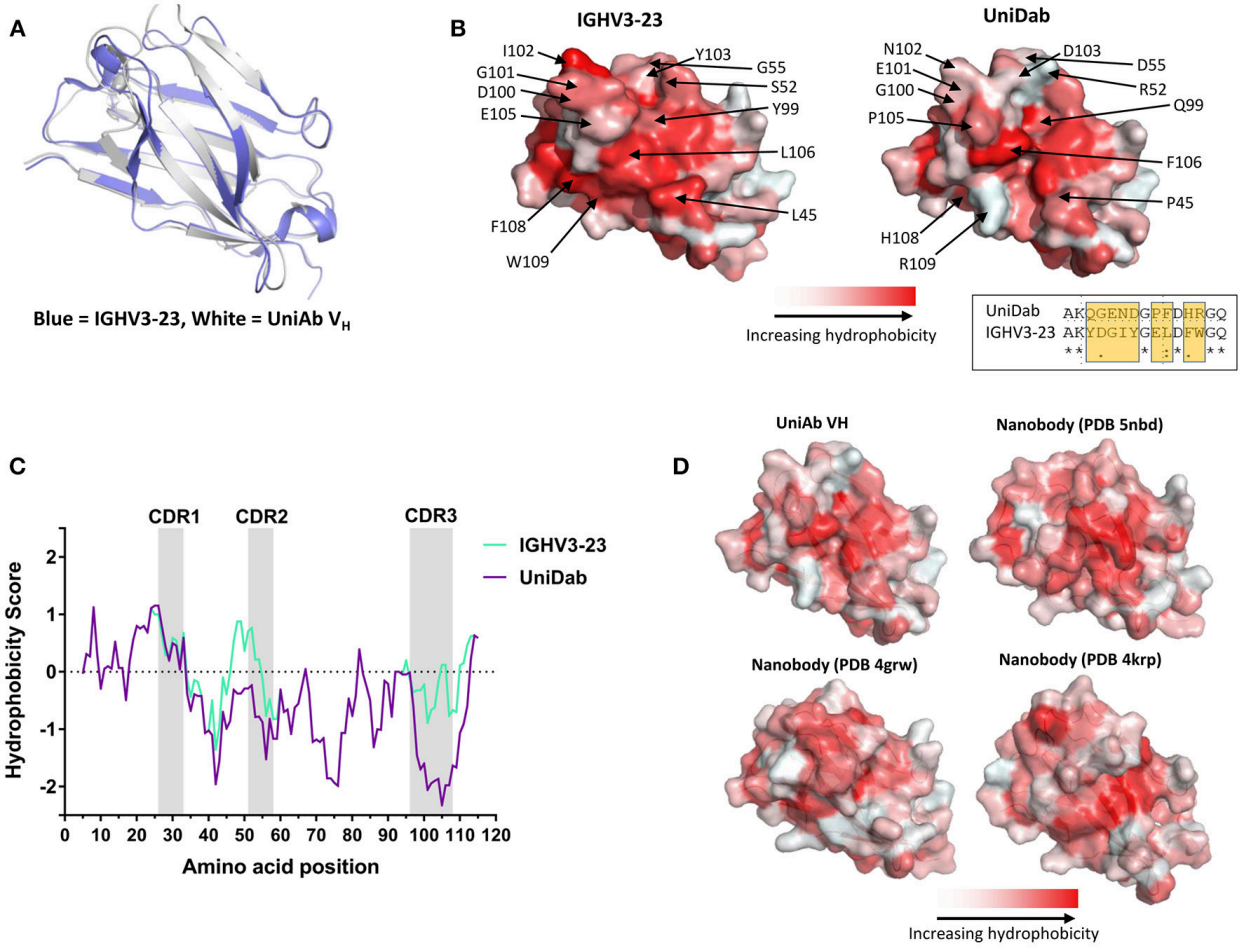
A UniAb V_H_ adopts a structure characteristic of conventional human V_H_ domains but displays reduced surface hydrophobicity. An anti-BCMA UniAb V_H_ was crystallized in complex with its antigen and the structure was solved. An overlay of the UniAb V_H_ (white) compared to a conventional human heavy chain of the same V gene (blue, PDB 5I1D) reveals highly similar 3-dimensional structures **(A)**. Mutations within the CDR2 and CDR3 as well as the framework mutation W101R (R109 in this V_H_) contribute to reduced hydrophobicity of the former light chain interacting surface in the UniAb V_H_ structure as compared to the conventional V_H_ **(B)**. An alignment of residues 97–111 is shown in the inset of **(B)**, highlighting the region with the greatest difference in hydrophobicity between the two molecules **(B)**. A Kyte-Doolittle plot of hydrophobicity illustrates the difference in sequence hydrophobicity proximal to the CDR2, CDR3, and framework 4 regions between the UniAb V_H_ and IGHV3-23 domain **(C)**. The surface hydrophobicity of the anti-BCMA UniAb V_H_ is similar to naturally occurring single domain antibodies derived from llamas (nanobody structures sourced from the PDB: 5NBD, 4GRW, and 4KRP) **(D)**.

Subsequently, we queried the PDB for similar human V_H_s for comparison with our UniAb V_H_ structure. We identified a human IgHV3-23 germline antibody (PDB 5I1D) that shared 87% sequence identity with our UniAb V_H_ across its framework 1–4 region. An overlay with our structure produced a RMSD of 1.5 Å, indicating that the UniAb V_H_ adopts a similar conformation to the conventional V_H_ (Figure 5A). Strikingly, a comparison of the light chain binding surface of the H2L2 V_H_ with the equivalent region of the UniAb V_H_ revealed reduced hydrophobicity due to several hydrophilic amino acid substitutions within the CDR2 and CDR3 regions as well as the W101R framework 4 mutation (W109R in this V_H_) (Figure 5B). A Kyte-Doolittle hydrophobicity plot highlights the role of these mutations in reducing the hydrophobicity of the UniAb V_H_ compared to the conventional V_H_ domain (Figure 5C). These results were recapitulated with comparison to a second human V_H_ structure (PDB 6AZM) (data not shown).

Next, we identified structures of three llama V_H_Hs with similarity to our UniAb sequence (PDB 5NBD, 4KRP, and 4GRW). Overlays of each structure with our UniAb V_H_ produced RMSD values of 3.61, 3.06, and 1.64 Å, respectively. Interestingly, the surface hydrophobicity of the UniAb V_H_ was similar to that of the llama V_H_Hs despite the UniAb lacking the hallmark framework 2 mutations known to increase the hydrophilicity of camelid V_H_Hs (Figure 5D) (1, 15, 39, 40). Taken together, these data suggest that antigen-specific UniAbs fold identically to classic human H2L2 V_H_ domains but may exhibit a hydrophobicity profile more closely aligned to naturally evolved HCAbs rather than that of conventional human V_H_ domains.

## Discussion

It is well-established that conventional human antibody repertoires can be expressed in engineered rodents, and furthermore that HCAbs are spontaneously produced if both C_H_1 domains and light chains are absent (17, 22, 41). We built on these results in the creation of UniRats, animals which lack all endogenous Ig expression and efficiently produce chimeric human/rat IgH molecules containing human V_H_, D, and J_H_ sequences on rat constant regions deleted for C_H_1. Although IgM and IgD are not synthesized due to the absence of Cμ and Cδ sequences, IgG2a is abundant and near normal numbers of B cells are observed within the bone marrow, spleen and lymph nodes. This implies that Cγ2a is sufficient for conventional differentiation events leading to extensive B cell development and likely culminating in expansion of plasmablasts. A further indication that the UniRat immune response is functioning as expected is that antigen-specific UniAbs show comparable somatic hypermutation rates to light chain containing antibodies derived using similar immunization strategies (22). Robust B cell development in UniRats is likely facilitated by the use of the chimeric human/rat IgH locus, which ensures a vigorous immune response by preserving interactions between the Ig molecules and the rat B cell signaling machinery (29, 31).

With the exception of Heavy Chain Disease, natural human antibodies are requisite heterodimers, composed of both heavy and light chains (42). Thus, it was initially unclear whether V_H_s from light chain-containing antibodies could be readily adapted to HCAb format. Indeed, attempts by several groups resulted in HCAbs with poor colloidal stability due to exposure of hydrophobic residues previously shielded by light chain binding (43, 44). However, our study of 124 purified UniAbs exhibiting robust expression and consistent colloidal and thermostability shows that HCAbs selected during *in vivo* maturation in a light chain-free animal represent a different class of molecule. Furthermore, in our analysis of over 2,000 UniAbs from 10 different immunization campaigns we report that on average only 5% associate with lambda light chain. Additional analyses reveal that a major contribution to the production of stable UniAbs comes from a single framework mutation, W101R, which plays a pivotal role in reducing the tendency of UniAbs to associate with light chains. By incorporating the W101R mutation into only the J4 gene, we were able to directly compare between UniAbs containing the W or the R variant, revealing that approximately 9 out of 10 UniAbs that associate with lambda light chain exhibit the wild type W variant at this position. Given these findings, we are currently developing new UniRat strains containing the W101R mutation in all six J genes. An intriguing question is whether the W101R mutation also prevents heavy chain association with the rat surrogate light chain, as endogenous V_preB_ and λ_5_ genes have not been silenced. Assuming a pre-B cell receptor configuration shapes the antibody response, a diminished or biased heavy-chain repertoire could be expected if this interaction were abrogated. This may indeed be the case, as we observe that certain V_H_ genes, such as V3–23 are more frequently expressed. However, this result can also be explained by the possibility that V_H_ selection influences later stages of B cell development or that not all human V_H_ sequences are equally suitable for expression as HCAbs. Furthermore, natural human antibody repertoires have been shown to exhibit biased V gene usage for reasons that are not fully understood (35, 36).

We note that the W101R mutation alone is neither necessary nor sufficient for abolishing light chain association. This finding concurs with work by other groups showing that mutations at several locations within a human V_H_, including at position 101, can result in stable human HCAbs that do not interact with light chains (33, 44). Although our analysis did not identify additional specific mutations apart from W101R that are generally predictive of light chain binding, we did uncover a statistically significant increase in the frequency of charged amino acids in the CDR2 and CDR3 loops of UniAbs as compared with H2L2 antibodies. It is possible that this is the result of selection within the UniRat favoring UniAbs with increased hydrophilicity, potentially to compensate for the absence of a light chain. While an intriguing result, this analysis focused on only two V genes, in future experiments we will extend this study to other V genes to further refine our understanding of amino acid usage in UniAbs.

The insights we obtained regarding the role of the W101R mutation as well as our observation of an increased frequency of hydrophilic residues in the CDR2 and CDR3 loops in UniAbs were further supported by crystallization of a UniAb V_H_ recognizing human BCMA. Analysis of this structure revealed that the W101R mutation (R109 in this V_H_) together with several hydrophilic amino acid substitutions within the CDR2 and CDR3 loops greatly reduced the surface hydrophobicity of the former light chain interface, while preserving the 3-dimensional fold of the V_H_. Interestingly, we observed that the surface hydrophobicity of the former light chain interface was similar to that of the corresponding region of several llama V_H_H sequences, though the UniAb V_H_ did not contain the hallmark camelid framework 2 mutations. Although these results represent a limited data set, they agree with our prediction that natural selection within the UniRat produces HCAbs that are well-adapted to function without light chains. In future studies we will complete structures of additional UniAb variable domains to build a more comprehensive understanding of surface hydrophobicity and the sequence-structure relationship within these molecules.

In summary, we describe here the first construction of a genetically engineered rat expressing human V_H_DJ_H_ genes in the absence of a C_H_1 domain, light chain, or endogenous antibodies. These animals mount robust immune responses and produce diverse repertoires of stable, high-affinity human HCAbs in response to antigen challenge. Successful production of UniAbs is likely influenced by several important features of the UniRat strains including the complete absence of light chain expression *in vivo*, introduced, and evolution-induced sequence mutations, and the use of a chimeric human/rat IgH locus. During high-throughput gene assembly human Fc sequences are easily substituted for the rat constant regions, so that screening and development proceeds with fully human sequences. Alternatively, the UniAb V_H_ regions may be expressed and used as single domain antibodies, facilitating even greater flexibility for development of novel antibody-based therapeutics. These molecules exhibit the favorable developability traits historically associated with conventional H2L2 antibodies, while providing the components for simple construction of fully human multispecific antibodies. Future studies will examine the utility of UniAbs for expediating the development of multispecific antibody-derived biologics.

## Materials and Methods

### Development of UniRat Transgenic Animals

Previously identified, characterized and modified BACs and YACs accommodate human heavy chain variable region genes and rat constant region genes (29, 31). To enable heavy-chain antibody expression, a rat constant region BAC was reassembled by replacement of Cμ and adjacent 3' regions with Cγ2α as the first C gene followed by Cγ1 and Cγ2b; all with C_H_1 exons removed. Locus functionality was retained by including 5' and 3' control regions with the downstream hs elements (3'αE) following Cε and Cα in the natural configuration. Heavy-chain-only expression was enforced by silencing of the endogenous heavy and light chain (kappa and lambda) loci. Full methods are detailed in Supplemental Methods File 1.

### B Cell Flow Cytometry

Spleens were harvested from wild type rats or UniRats and cell suspensions prepared. Red blood cells were removed by standard erythrocyte lysis. Cells were then stained with Pacific Orange conjugated mouse anti-IgM (Jackson ImmunoResearch), Alexa-647 conjugated mouse anti-rat IgD (MARD-4), FITC conjugated mouse anti-rat IgG1, anti-rat IgG2a, or anti-rat IgG2b (BD Biosciences), PE-conjugated mouse anti-rat CD45R (clone His24, BD Bioscience), and PerCP conjugated mouse anti-rat TCR (clone R7.3, BD Biosciences) monoclonal antibodies for 30 min at 4°C. Stained cells were run on a FACS Verse Cytometer (BD Bioscience) and the data were analyzed with FlowJo Software (Tree Star).

### Purification and Gel Electrophoresis of UniRat Ig

Protein A agarose was used to purify rat IgG (Innova Biosciences, Cambridge, UK, product numbers: 851-0024). Rat serum was incubated with resin in a buffer containing 0.1 M sodium phosphate pH 8 (protein A) with gentle shaking. The mixture was washed thoroughly with PBS (pH 7.4), followed by elution with 0.1 M sodium citrate (pH 2.5) and neutralized with 1 M Tris-HCL (pH 9). Gel electrophoresis was conducted using 4–15% SDS-PAGE and stained with Coomassie brilliant blue. The HyperPAGE prestained protein marker (BIO-33066; Bioline) was used as the molecular weight standard.

### Immunizations, Next-Generation Sequencing, Clonotype Analysis, and Cloning

Methods essentially as described in Harris et al. (22). In brief, UniRat or OmniFlic (Ligand Pharmaceuticals, San Diego, CA) animals were immunized using standard adjuvants along with recombinant protein antigens. Plasma samples were collected post-immunization to assess serum titers against the antigen by ELISA. After approximately 7 weeks of immunization, draining lymph nodes were harvested and total RNA was isolated. Ig heavy chain sequences were amplified using first strand cDNA synthesis and 5′ RACE by PCR, following methods similar to those previously described (45, 46) and then purified by gel extraction. Next-generation sequencing (NGS) was completed using the MiSeq platform (Illumina) with 2 × 300 paired-end reads. To enable multiplexing of samples, indexing labels were added by primer extension (47). Approximately 100,000 paired reads covered each sample, and those that showed alignment of <20 nucleotides to a human Ig locus were discarded. Merged forward and reverse reads of V_H_ regions were translated into open reading frames and framework and CDR regions identified by IGBLAST (https://www.ncbi.nlm.nih.gov/igblast/). Clonotypes [defined by CDR3 protein sequences with at least 80% sequence similarity (48, 49)] were determined for samples using agglomerative clustering. CDR3 clonotypes were ranked by the percent of total reads in a sample defined by that clonotype. Those with the greatest abundance were prioritized for high-throughput cloning into an expression vector containing a C_H_1-deleted human IgG1 Fc region and validated by Sanger sequencing. Plasmids were transformed into *E. coli* grown in LB culture media and then purified to enable transient transfection of HEK293 cells in 96-well format. Following several days of expression, supernatants containing antibody were harvested and clarified by centrifugation.

### High Throughput ELISA

Methods essentially as described (22, 45). Briefly, recombinant proteins corresponding to the antigen(s) for each immunization campaign were coated overnight at 4°C in 96-well plates using BupH Carbonate-Bicarbonate buffer. Plates were then washed with TBST (20 mM Tris, 150 mM NaCl, 0.05% Tween-20, pH 7.6) and blocked with blocking buffer (TBST with 1% dry milk powder). HEK293 supernatants containing antibodies were diluted 1:100 in blocking buffer and added to antigen-coated plates. Detection of bound antibodies was accomplished using an HRP-labeled anti-human Ig secondary antibody together with chemiluminescent substrate. Luminescence was quantified (SpectraMax i3X, Molecular devices) and the signal for each well was normalized by dividing by the average background luminescence of antigen-coated wells that had been incubated with supernatant from untransfected HEK293 cells. A minimum of 30-fold over background was required to assign a well as ELISA-positive.

### IGHV Gene Usage Analysis

Ig heavy chain variable gene usage was calculated as previously described (22). In summary, UniAb amino acid sequences were aligned to the germline reference sequences sourced from IMGT. The percentage of UniAbs in a given sample that used each IGHV-gene was determined and calculated the averaged across multiple samples (55 HC31 animals, 79 HC27 animals). The limit of detection was defined by the frequency below which matches to IGHV-genes not present in the UniRat genomes were reported.

### Somatic Hypermutation Analysis

SHM was determined as detailed in Harris et al. (22). Briefly, we aligned each IGHV amino acid sequence with the germline reference sequence sourced from IMGT and calculated the total number of mismatches. UniAbs were then categorized as lambda binding positive or antigen binding positive by ELISA and binned based on the total number of mismatches from germline (0, 1, 2, etc.), then the number in each bin was converted to a percent value. GraphPad Prism V7 software was used to generate graphs.

### UniAb Purification and SEC-UHPLC

Soluble UniAb was expressed in CHO cells using protein free media supplemented with L-Glutamine (Invitrogen). Cell harvest was clarified by centrifugation and 0.2 μm PES filtration. During the first purification, step the clarified supernatant was loaded on a MabSelect SuRe (GE#17543801) resin, washed with PBS, followed by elution with 50 mM Acetic Acid, 10% Glycerol, 10% Sucrose, pH 3.6. The elution pool was immediately neutralized to approximately pH 6.5 with 2 M Tris, pH 9. After the first capture step, the protein pool was concentrated and loaded on to a Superdex 200i 10X300GL column (GE#28990944) to remove any high molecular weight species. Fractions were collected, and the most homogenous fractions were pooled. UniAb protein pools were analyzed for aggregation and purity by SEC-UHPLC. A ThermoFisher UltiMate^TM^ 3,000 UHPLC system was used with a TSK-Gel UP-SW3000 (Tosoh#23449) column in line. The mobile phase was 100 mM Citrate, 500 mM NaCl, 200 mM Arginine, pH 6.2 run at a flow rate of 0.25 ml/min and the method was run in 100% A isocratic mode. Fifty μg of protein was injected and 280 nm UV absorbance monitored over the 10 min run. The UV trace was analyzed and integrated by area under the curve to determine percent aggregation, monomer and degradants.

### UniAb Affinity Measurements by Biolayer Interferometry (Octet)

All measurements were carried out on an Octet Qk384 instrument (Pall). In a typical experiment, sensors were loaded with 5–10 μg/mL UniAb using the AHC sensor (Pall). Antigens were either expressed in-house (ECD with His-tag) or commercially available. Kinetic parameters k_on_ and k_off_ were measured in the presence of increasing concentrations of each antigen, spanning a 0.1 to 10 x Kd range. Association and dissociation curves obtained in the sensorgrams were fitted to a 1:1 binding model using the instrument's data analysis software package v9.0.

### Differential Scanning Calorimetry (DSC)

Purified antibodies at a concentration of 1 mg/mL in buffer (20 mM citrate, 100 mM NaCl, pH 6.2) were submitted to the Nano DSC system (TA instrument) for analysis. A temperature ramp of 1°C/min was performed with monitoring from 25 to 100°C. Thermograms of the blank buffer were subtracted from each antibody prior to analysis and the Tm values were calculated after deconvolution using the Nano DSC software.

### Bioinformatics Analysis of Amino Acid Usage

Each amino acid was assigned to one of the following four classes: polar uncharged—S, T, N, Q, charged—R, H, K, D, E, hydrophobic—A, V, I, L, M, F, Y, W, special cases—C, G, P. For a given antibody, we counted the total number of residues assigned to a given class within each of the three CDRs and then divided this sum by the total length of each individual CDR to obtain a frequency metric in the interval [0, 1]. Thus, for each antibody, we computed a total of 12 frequencies (3 CDR regions x 4 amino acid classes). In total 4,275 and 3,296 UniAbs, using the V-genes IgHV3-23 and IgHV4-39, respectively, were analyzed and compared against 2,617 IgHV3-23 and 1,598 IgHV4-39 H2L2 antibodies. The UniAb sequences were sourced from 30 separate immunization campaigns, while the H2L2 antibodies were derived from 32 distinct immunizations of transgenic rats expressing human antibodies with fixed light chains. Within a V-gene group, we compared the set of calculated frequencies for all four amino acid classes, for each distinct CDR (12 comparisons in total). We ran a series of two-sided Mann–Whitney–Wilcoxon (aka Mann-Whitney U) statistical tests utilizing the open-source software SciPy statistical package (version 1.1.0; http://scipy.org). Specifically, we ran the function “scipy.stats.stats.mannwhitneyu” from the SciPy package, with the “alternative” argument to the function set to “two-sided”. We executed each test under the null hypothesis assertion that there is no difference between amino acid class frequencies for UniAb antibodies vs. H2L2 antibodies within each CDR. The frequencies in each category were compared between UniAb and H2L2 antibodies by subtracting each H2L2 frequency from the corresponding UniAb frequency and converting the decimal to a percent. Jones E, Oliphant E, Peterson P, et al. SciPy: Open Source Scientific Tools for Python, 2001-, http://www.scipy.org/ [Online; accessed 2018-07-31].

### Crystallography

Complexes of a UniAb V_H_ (V_H_3-23) with its antigen, the extracellular domain of human BCMA, were formed by mixing BCMA:UniAb V_H_ at a 1:2 molar ratio for 20 min at 4°C, followed by isolation with size exclusion chromatography using 20 mM PBS, 250 mM NaCl running buffer. Crystallization was carried out in a sitting drop vapor diffusion method. Crystals appeared in a condition containing 0.2 M sodium malonate pH 4.0 and 20% PEG 3350. Crystals were soaked in mother liquor containing 25% ethylene glycol before flash freezing in liquid nitrogen for synchrotron data collection. Diffraction data was collected on the MX2 beamline at the Australian Synchrotron. XDS package (50) was used for initial indexing of the reflections. Aimless module from CCP4i was used for subsequent scaling and merging of the intensities. Molecular replacement was performed using the Phaser (51) module of CPP4i suite with search models Protein Data Bank (PDB) entries 3ZHK (chain A) and 4ZFO (chain F) for the V_H_ and BCMA, respectively. A molecular replacement solution could be found with two molecules of UniAb V_H_-BCMA complex in the asymmetric unit. Structure was then manually inspected and corrected using Coot (52), and several cycles of refinement were performed using REFMAC (53) until the R factors converged. The complete data collection and refinement statistics are given in Supplemental Table 3.

### Structure Comparison and Analysis

Sequence alignments were generated using ClustalX (54). Structure figures were created using PyMOL (The PyMOL Molecular Graphics System, Version 2.0, Schrödinger, LLC.). Structures were superimposed/structurally aligned using the “super” command implemented within PyMOL. For visualization of the general hydrophobicity characteristics, proteins were colored according to the normalized consensus hydrophobicity scale (55) as implemented by the color_h script within PyMOL.

### Kyte-Doolittle Plot

Framework 1–4 amino acid sequences were entered into the ProtScale tool of ExPasy (56) with the Kyte & Doolittle (57) hydrophobicity scale option selected. The window size was set to 9 and a linear weight variation model was used. The results were obtained in minimal numerical format to allow for visualization of plots using GraphPad Prism V7 software.

## Ethics Statement

This study was carried out in a research facility registered with the California Department of Public Health and, as such, the animal care and use program is required to adhere to the NIH Guide for the Care and Use of Laboratory Animals. We certify that all work was performed using Institutional Animal Care and Use Committee (IACUC) protocols that have been reviewed and approved by our IACUC committee.

## Author Contributions

BM, MO, MB, and RB created the UniRat strains; flow cytometry was conducted by L-HO. NGS and molecular biology was managed by KH. ELISAs were conducted by AaB, SC, KD, HO, DP, and PS. antibody purification, affinity and stability analysis were completed by LD, BJ, PP, and HU. AnB and NT performed bioinformatics. Experiment conception, design, and data analysis was conducted by NT, US, SC, IA, WvS, RB, and SF. Figures were generated by SC, L-HO, MB, and NT. The manuscript was written by SC and SF, all authors read and approved the submitted version.

### Conflict of Interest Statement

All authors are employees of Teneobio Inc with equity interests or paid consultants of Teneobio Inc. Teneobio owns and has filed patents pertaining to the generation of UniAbs and fixed-light chain antibodies.
