# Supplementary material for: Multispecific Antibody Development Platform Based on Human Heavy Chain Antibodies
Source: Front Immunol. 2019 Jan 7;9:3037. doi: 10.3389/fimmu.2018.03037 (PMC6330309; doi:10.3389/fimmu.2018.03037)
Supplement: Supplementary file 1 [file Image_1.pdf]

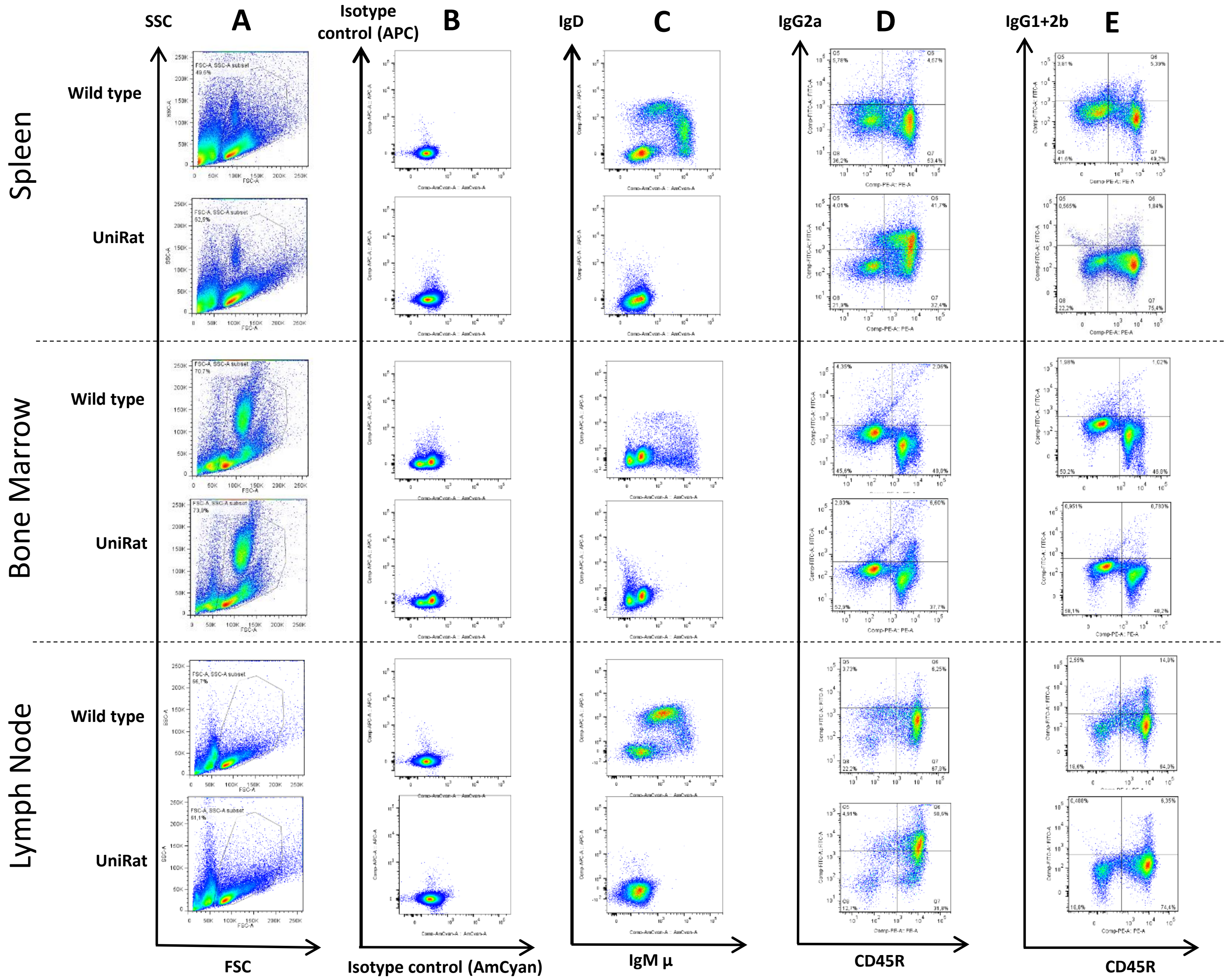

Supplemental Figure 1. Characterization of B cells isolated from the spleens, bone marrow and lymph nodes of UniRats compared to wild type rats after immunization with β-galactosidase. Data from a representative wild type and UniRat animal are shown. (A) Forward and side scatter, (B) Isotype controls for anti-IgD and anti-IgM staining, (C) IgD and IgM are not produced by UniRats, (D) IgG2a+ B cells are produced at higher numbers in UniRats, (E) IgG1+ and IgG2+ B cells are present at equivalent or slightly lower levels in UniRats compared to wild type rats. Group size of n=4 analyzed.
