# Supplementary material for: Multispecific Antibody Development Platform Based on Human Heavy Chain Antibodies
Source: Front Immunol. 2019 Jan 7;9:3037. doi: 10.3389/fimmu.2018.03037 (PMC6330309; doi:10.3389/fimmu.2018.03037)
Supplement: Supplementary file 2 [file Image_2.pdf]

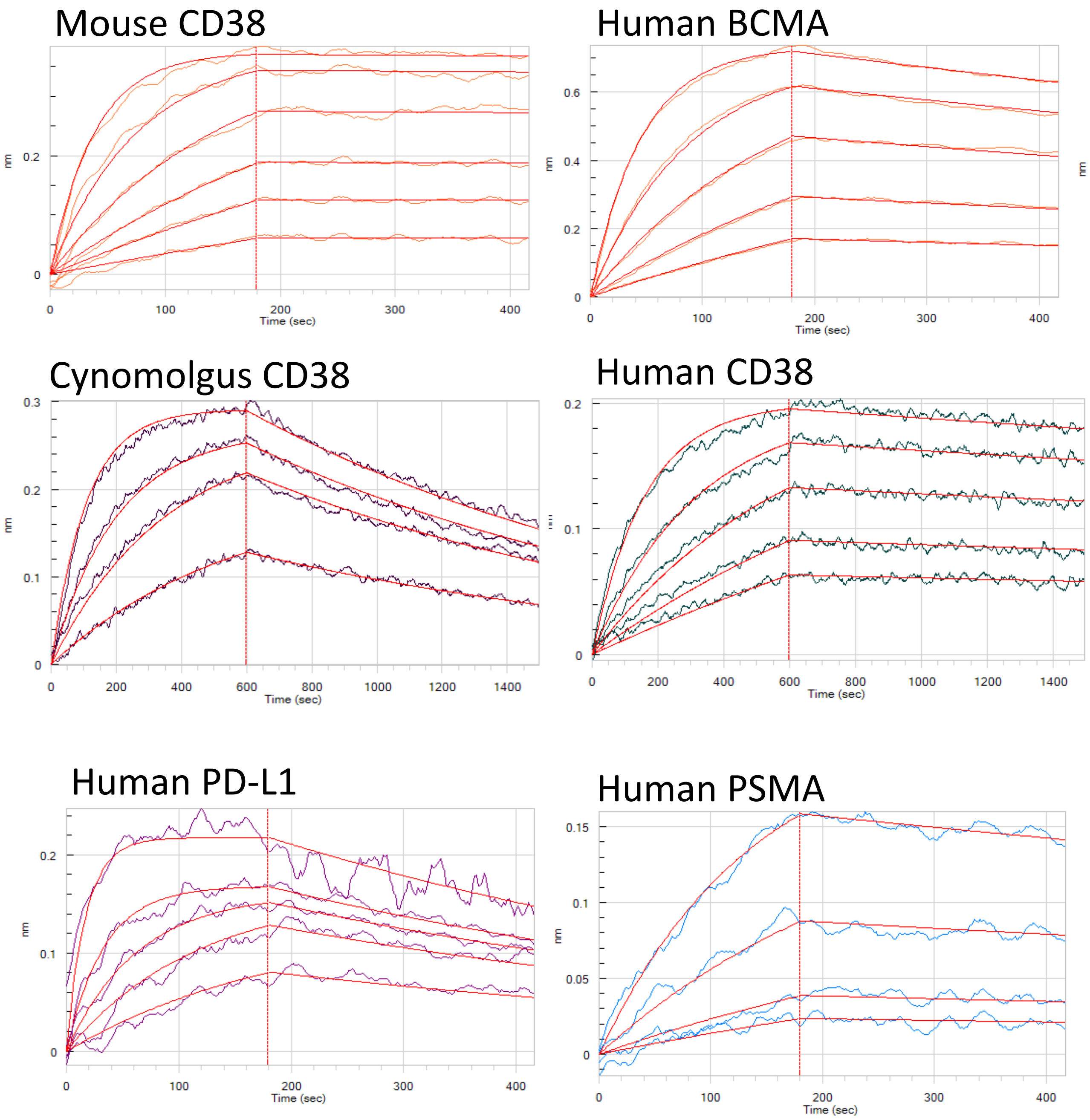

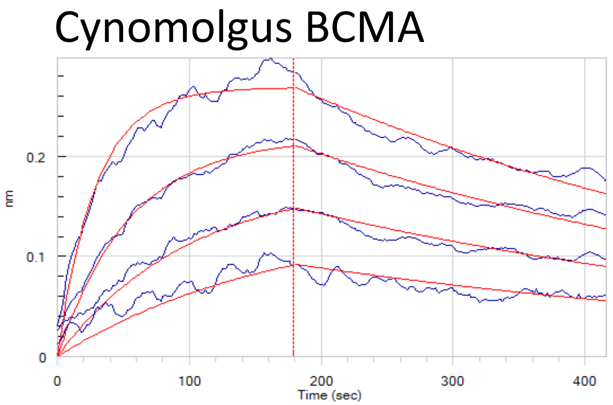

Sample	KD (M)	kon (1/Ms)	kdis(1/s)	Full R^2	Highest antigen concentration tested (nM)
Mouse CD38	2.17E-10	1.65E+05	3.58E-05	0.996	160
Human BCMA	3.43E-10	1.67E+06	5.73E-04	0.999	13
Cynomolgus CD38	1.02E-09	6.89E+05	7.01E-04	0.993	100
Human CD38	2.55E-09	3.33E+04	8.47E-05	0.990	100
Human PD-L1	3.63E-09	4.54E+05	1.65E-03	0.970	500
Human PSMA	7.06E-09	6.80E+04	4.80E-04	0.991	100
Cynomolgus BCMA	8.27E-09	2.58E+05	2.13E-03	0.979	500

Supplemental Figure 2. Biolayer interferometry (Octet) sensorgrams and kinetic parameters for individual UniAbs isolated from primary screens. Data corresponds to the KD values reported in Table 3. Two-fold serial dilutions of each antigen were tested. The highest antigen concentration tested for each antibody is indicated in the above table.
