# Supplementary material for: Multispecific Antibody Development Platform Based on Human Heavy Chain Antibodies
Source: Front Immunol. 2019 Jan 7;9:3037. doi: 10.3389/fimmu.2018.03037 (PMC6330309; doi:10.3389/fimmu.2018.03037)
Supplement: Supplementary file 3 [file Image_3.pdf]

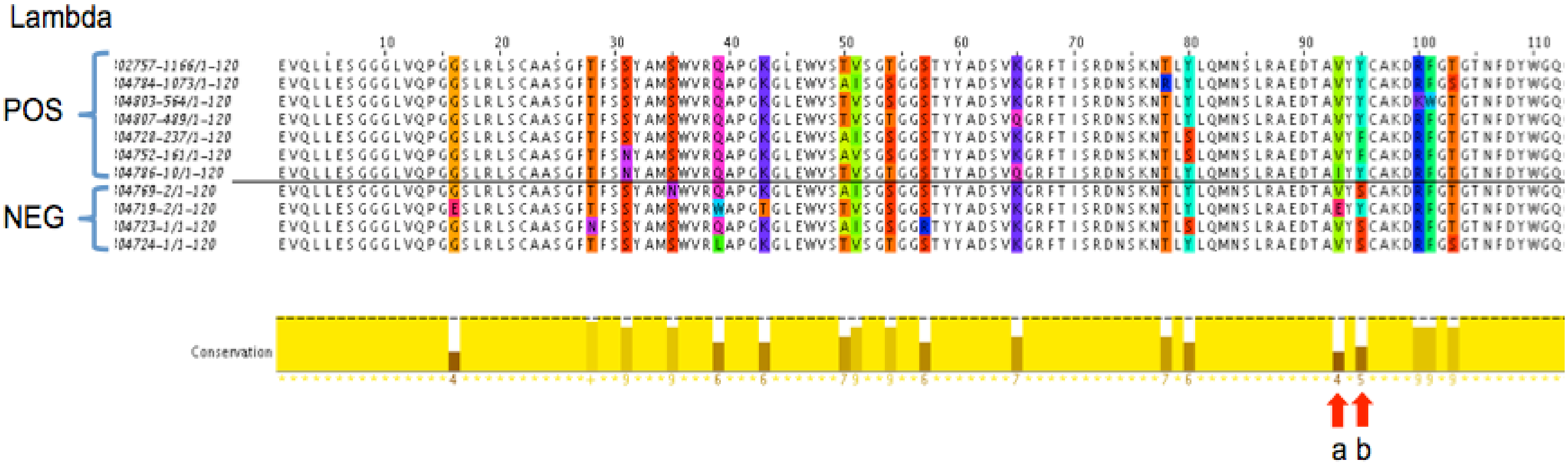


**Supplemental Figure 3. Lambda protein association with heavy chain UniAbs in the same Clonotype**. A multiple sequence alignment of 11 $V_H$ sequences from heavy chain antibodies in the same CDR3 family is shown. All sequences contain a W at the framework 4 position 101 (position 110 in this alignment). The top 7 sequences in the alignment are positive for lambda association as measured by ELISA, while the bottom 4 sequences in the alignment are negative for lambda association. This clonotype family displays additional mutations indicated by an **a** and **b** that discriminate between the lambda positive and negative sequences. A Glu at position a or a Ser at position b eliminates lambda association. However, Ser or Glu at these positions in other clonotypes did not have the same association with lambda binding. Results from this family suggest that there are compensatory mutations in some W101 $V_H$ sequences that prevent lambda association, but that these compensatory mutations may be specific to a particular clonotype.
