# Supplementary material for: Multispecific Antibody Development Platform Based on Human Heavy Chain Antibodies
Source: Front Immunol. 2019 Jan 7;9:3037. doi: 10.3389/fimmu.2018.03037 (PMC6330309; doi:10.3389/fimmu.2018.03037)
Supplement: Supplementary file 4 [file Table_1.pdf]

Supplemental table 1. Differential scanning calorimetry was used to determine melting temperatures for the V_H and C_H2 and C_H3 domains of six UniAbs. The melting temperatures of the UniAb V_H domains were 61.8 °C on average.

	DSC Observed Transition		
**Identifier**	**Tm: V_H**	**Tm: C_H2**	**Tm: C_H3**
**316274**	59.1	66.3	69.3
**316833**	59.2	64.9	69
**308902**	61.7	65.9	68.9
**316232**	61.8	64.1	67.8
**308912**	61.9	66	68.6
**316829**	66.8	68.1	80.8
