# Supplementary material for: Multispecific Antibody Development Platform Based on Human Heavy Chain Antibodies
Source: Front Immunol. 2019 Jan 7;9:3037. doi: 10.3389/fimmu.2018.03037 (PMC6330309; doi:10.3389/fimmu.2018.03037)
Supplement: Supplementary file 5 [file Table_2.pdf]

Supplemental table 2. Amino acid usage frequencies and standard deviations in UniAbs and H2L2 antibodies. Amino acids were categorized into one of four classes: hydrophobic, special cases, charged and polar uncharged. The frequency of each case in the CDR1, CDR2 and CDR3 was determined for a large set of UniAbs and H2L2 antibodies that used either IGHV3-23 or IGHV4-39. The difference in frequencies for each category were then compared between UniAbs and H2L2 antibodies by subtracting each H2L2 frequency from the corresponding UniRat frequency and converting the calculated value to a percent. A Mann-Whitney test was conducted in cases where more than a 2% difference was found (highlighted).

	CDR_category	H2L2: Avg Frequency	H2L2: Std_Dev	Unirat: Avg Frequency	Unirat: Std Dev	% Difference: UniRat vs H2L2	p-value
V3-23	CDR1: hydrophobic	0.487	0.06	0.485	0.05	-0.2	
	CDR1: special cases	0.136	0.05	0.134	0.03	-0.3	
	CDR1: charged	0.021	0.05	0.011	0.04	-1.1	
	CDR1: polar uncharged	0.355	0.07	0.370	0.05	+1.6	
	CDR2: hydrophobic	0.158	0.07	0.143	0.05	-1.5	
	CDR2: special cases	0.343	0.10	0.336	0.08	-0.6	
	CDR2: charged	0.039	0.07	0.043	0.08	+0.4	
	CDR2: polar uncharged	0.460	0.09	0.478	0.07	+1.7	
	CDR3: hydrophobic	0.481	0.10	0.394	0.10	-8.7	1.3E-223
	CDR3: special cases	0.166	0.09	0.168	0.09	+0.1	
	CDR3: charged	0.233	0.11	0.283	0.10	+5.0	2.7E-87
	CDR3: polar uncharged	0.120	0.09	0.156	0.10	+3.6	1.4E-44

	CDR_category	H2L2: Avg Frequency	H2L2: Std_Dev	Unirat: Avg Frequency	Unirat: Std_Dev	% Difference: UniRat vs H2L2	p-value
V4-39	CDR1: hydrophobic	0.310	0.06	0.303	0.04	-0.7	
	CDR1: special cases	0.198	0.06	0.198	0.05	0.0	
	CDR1: charged	0.041	0.06	0.037	0.06	-0.4	
	CDR1: polar uncharged	0.452	0.07	0.463	0.07	+1.1	
	CDR2: hydrophobic	0.417	0.06	0.398	0.09	-1.9	
	CDR2: special cases	0.150	0.04	0.147	0.04	-0.3	
	CDR2: charged	0.022	0.06	0.046	0.08	+2.4	1.0E-24
	CDR2: polar uncharged	0.411	0.07	0.409	0.08	-0.2	
	CDR3: hydrophobic	0.458	0.10	0.384	0.11	-7.4	1.4E-101
	CDR3: special cases	0.170	0.08	0.151	0.09	-1.9	
	CDR3: charged	0.248	0.10	0.282	0.09	+3.4	1.6E-29
	CDR3: polar uncharged	0.124	0.10	0.182	0.11	+5.8	1.2E-65
