# Supplementary material for: Multispecific Antibody Development Platform Based on Human Heavy Chain Antibodies
Source: Front Immunol. 2019 Jan 7;9:3037. doi: 10.3389/fimmu.2018.03037 (PMC6330309; doi:10.3389/fimmu.2018.03037)
Supplement: Supplementary file 6 [file Table_3.pdf]

Supplemental table 3. Diffraction data and structure refinement statistics.

**Data collection**	**UniDab-BCMA**
Beamline	MX2, Australian Synchrotron
Resolution (Å)	48-2.59 (2.7-2.59)
Space group	C 2 2 21
Completeness (%)	100 (99.4)
**Cell dimensions**	
a, b, c (Å)	65.39, 73.80, 133.49
α, β, g (°)	90, 90, 90
I/□ I	12 (3.1)
R_{pim}	0.03 (0.10)
R_{merge}	0.07 (0.25)
**Refinement**	
Resolution (Å)	48-2.6 (2.66-2.6)
Total Number of reflections	9739
Total Number of Atoms	2361
Completeness (%)	99.77 (99.73)
Overall Correlation coefficient	0.94
R_{work}	0.20 (0.24)
R_{free}	0.28 (0.29)
**RMSD**	
Bond angles (°)	1.6
Bond length (Å)	0.012
**Ramachandran Plot analysis**	
Preferred regions (%)	96.08
Allowed regions (%)	3.92
Outliers (%)	0
