# Supplementary material for: Multispecific Antibody Development Platform Based on Human Heavy Chain Antibodies
Source: Front Immunol. 2019 Jan 7;9:3037. doi: 10.3389/fimmu.2018.03037 (PMC6330309; doi:10.3389/fimmu.2018.03037)
Supplement: Supplementary file 7 [file Data_Sheet_1.docx]

**Development of UniRat transgenic animals
The generation of genetically engineered rats expressing heavy chain-only antibodies (HC27 construction method)**

Previously identified, characterized and, in part, modified BACs and YACs accommodate human heavy chain variable region genes and rat constant region genes (Osborn et al., 2013; Ma et al., 2013). To enable heavy-chain antibody expression, a rat constant region BAC was modified by removal of Cµ and deletion of C_H_1 exons in all Cγs. Heavy-chain-only expression was then enforced by silencing of the endogenous heavy and light chain (kappa and lambda) loci.

### Construction of modified human IgH loci on YACs and BACs.

A ‘human – rat’ IgH locus was constructed and assembled in several parts. This involved the modification and joining of rat C region genes downstream of human J_H_s and subsequently, the upstream addition of the human V_H_6 –D-segment region. Two BACs with separate clusters of human V_H_ genes [BAC6 and BAC3] were then co-injected with the BAC termed Georg, encoding the assembled and modified region comprising human V_H_6 – all Ds – all J_H_s - rat Cγ2a/1/2b (ΔC_H_1).

For the introduction of modifications at precise locations in the DNA sequence and for simultaneously joining multiple large DNA regions, technologies were developed to assemble sequences with overlapping ends in *S. cerevisiae* as circular YAC (cYAC) and, subsequently, to convert such cYACs into BACs. Advantages of YACs include their capacity to retain large DNA inserts, the ease of homologous alterations in the yeast host and the maintenance of sequence stability especially in the highly repetitive regions (e.g. switch regions, enhancers). On the other hand BACs, propagated in *E. coli*, offer the advantages of easy preparation and large yield. In addition, detailed restriction mapping and sequence analysis can be better achieved from BACs than YACs. Two self-replicating *S. cerevisiae*/*E. Coli* shuttle vectors, pBelo-CEN-URA and pCAU were constructed. Briefly, *S. cerevisiae* CEN4 was cut out as an AvrII fragment from pYAC-RC (Marchuk and Collins, 1988) and ligated to SpeI–linearised pAP599 (Ma et al. Mol Microbiol. 2007 Oct;66(1):14-25). The resulting plasmid contains CEN4 cloned downstream of URA3. From this, an ApaLI–BamHI URA3 - CEN4 fragment was cut out and ligated to ApaLI and BamHI digested pBACBelo11 (New England Biolabs) to yield pBelo-CEN-URA. The *S. cerevisiae* autonomously replicating sequence ARS209 was synthesized and cloned into a unique SexAI site in pBelo-CEN-URA to yield pCAU.

To facilitate the modifications of human J_H_4, rat Cµ and Cγ1 regions, a ~ 37 kb SacII-fragment spanning from ~ 2.2 kb upstream of the human J_H_s to ~ 5.5 kb downstream of the rat Cγ1 coding region was cut out from the BAC construct Annabel (Osborn et al., 2013) and cloned into a unique SacII site in pBelo-CEN-URA [pBelo + SacII, 37 kb]. In addition, to modify the rat Cγ2b region, a ~ 19 kb SacII – SwaI fragment from Annabel spanning from ~ 6.9 kb upstream of the γ2b switch region to ~ 2.0 kb downstream of the Cγ2b coding region was cloned into SacII and HpaI – double digested pBelo-CEN-URA [pBelo + SacII – SwaI, 19 kb]. Both plasmids were used as templates for amplifying various human and rat genomic regions and to establish the required restriction fragments.

The DNA region spanning from ~ 3.1 kb upstream of the human J_H_s and including rat Cµ with some 3’ region was modified and assembled in pCAU as a 16.7 kb SnaBI – FspI fragment. The modified region includes all authentic human J_H_s except a T → C point mutation being introduced into J_H_4 (resulting into a W → R amino acid change) followed by the rat intergenic region from the J_H_s until µCH1, which was deleted along with the rest of Cµ coding region and replaced precisely by rat Cγ2a sequence lacking C_H_1 (starting from the intron immediately upstream of Hinge to the 3’ end of the membrane exons). This construct was derived by the assembly of the following 5 overlapping fragments in yeast as cYAC and then converted into a BAC: an amplified ~ 4.3 kb fragment using primers HC27 – 1 and – 2 covering the region upstream of human J_H_ to mutated J_H_4 (the point mutation introduced via the latter primer indicated by **G**), an amplified ~3.4 kb fragment using primers HC27 – 3 and – 4 spanning from mutated J_H_4 (indicated by **C**) to upstream of the µ switch region, a ~5.2 kb AflII – fragment encompassing the µ switch region and the flanking sequences cut out from pBelo + SacII 37 kb, an amplified rat Cγ2a lacking C_H_1 fused to sequences flanking rat Cµ using long primers HC27 – 5 and – 6, and amplification of the pCAU vector using primers HC27 – 7 and – 8. This resulted in pCAU + HuJ - Rat Cγ2a(-CH1). All modified regions were checked by sequencing to confirm the accuracy.

Rat Cγ1 lacking C_H_1 and Cγ2b lacking C_H_1 were individually generated via PCR. A ~1.7 kb fragment located immediately upstream of the Cγ1 coding region with a 3’ tail matching the 5’ end of the intron between C_H_1 and hinge was amplified using primers HC27 – 9 and – 10. Cγ1 was amplified as a ~ 3.9 kb fragment from the intron between C_H_1 and hinge to the 3’ end of the coding region using primers HC27 – 11 and – 12. Subsequently, the ~ 1.7 kb and ~ 3.9 kb fragments were both gel purified and joined via overlapping PCR using primers HC27 – 9 and – 12 to yield a ~ 5.6 kb fragment. Similarly, for Cγ2b without C_H_1, a ~ 0.3 kb fragment upstream of the Cγ2b coding region was amplified using primers HC27 – 13 and – 14, and a ~ 5.4 kb fragment - spanning the area from the intron between C_H_1 and hinge to the 3’ end of the coding region - was amplified using primers HC27 – 15 and – 16, and subsequently, these two fragments were joined via overlapping PCR using primers HC27 – 13 and – 16 to yield a ~ 5.7 kb fragment. pCAU + Rat Cγ1, 2b(-CH1s) was constructed to contain the following: 100 bp homology region matching the 3’ end of rat Cµ, followed by Cγ1 and Cγ2b in the genomic configuration except the C_H_1s of both were deleted. Six overlapping fragments were used to construct pCAU + Rat Cγ1, 2b(-CH1s): a ~ 10.2 kb SpeI – NarI fragment spanning from the 3’ Cµ homology region followed by the γ1 switch region cut out from pBelo + SacII, 37 kb, the ~5.6 kb PCR fragment containing Cγ1 without C_H_1 as described above, an amplified ~ 7.4 kb fragment covering the intergenic region between Cγ1 and Cγ2b using primers HC27 – 17 and – 18, a ~ 11.3 kb XhoI fragment encompassing the rat Cγ2b switch region cut out from pBelo + SacII – SwaI 19 kb, the ~ 5.7 kb PCR fragment containing Cγ2b without C_H_1, and the amplified pCAU vector using primers HC27 – 19 and – 20. The rat genomic region in pCAU + Rat Cγ1, 2b(-CH1s) can be cut out as a single ~ 40 kb FspI fragment.

Finally, the BAC (Georg) encoding the human V_H_6 -Ds - J_H_s-rat C regions with all the modifications was assembled using the following four overlapping fragments: a purified ~ 78.2 kb FspAI – MluI fragment encompassing the human V_H_6 –Ds region cut out from BAC10 (CTD-3216M13, Invitrogen), the 16.7 kb SnaBI – FspI fragment cut out from pCAU + HuJ - Rat Cγ2a(-CH1) as described above, the ~ 40 kb FspI fragment cut out from pCAU + Rat Cγ1, 2b(-CH1s), and a purified ~ 77.2 kb SwaI – SacII fragment cut out from construct Annabel which includes the intergenic region between Cγ2b and C_Ɛ_ followed by C_Ɛ,_ C_α,_ the 3’ enhancer region, the pBelo-CEN-URA vector, and the 5’ region upstream of human V_H_6. This final construct was checked extensively via restriction mapping and partial sequencing. The (human V_H_6 -Ds - J_H_s-rat C) region can be cut out and purified as a ~ 201 kb NotI fragment.

BAC6 contains the human genomic region from VH4-39 to VH3-23, while BAC3 contains a downstream region from VH3-11 to VH6-1 (the most D proximal VH gene). To provide an overlap between BAC6 and BAC3, a 10.6 kb fragment located at the 5’ end of the human V_H_ loci in BAC3 was integrated downstream of VH3-23 in BAC6 as described previously (Osborn et al. 2013). The human V_H_ genes in BAC6 were cut out as a ~182-kb AsiSI -AscI fragment. BAC3 was unmodified and the human V_H_ genes in this BAC were cut out as a ~ 173 kb NotI – fragment.

Oligonucleotides:
HC27 – 1: GTATTACACACAAAATGGGAAAAGCTG
HC27 – 2: CC**G**GTAGTCAAAGTAGTCACATTGTGGGAGGC
HC27 – 3: CCTTAATGGGGCCTCCCACAATGTGACTACTTTGACTAC**C**GGGGCCAGGGAACCCTGGTCACCG
HC27 – 4: GAATCCTAGGATTGCCTTCTTAGCCTG
HC27 – 5: CCATAGACCAAACTTACCTACTATCTAGTCCTGCCAACCTTAAGAGCAGCAACATGGAGACAGCAGAGTGTAGAGAGATCTCCTGACTGGCAGGAGGCAAGAAGATGGATTCTTACTCGTCCATTTCTCTTTTATCCCTCTCTGGTCCTAGAGAACAACCAGGGGATGAGGGGCTC
HC27 – 6: GCACAAGTGGACAAAGTCTTTGGCCAGTCTAGAAAGAAGCCCGTCTCAGAGATCAAAGCTGGAGGGCAACACAGGAAAGATGTGGGAATAAGTTTACTAGTCATACAGGCAGGAACCCCAGGCCCAGAGGTAGTGTCCCTGTGGGAGGGTCTCTTGCTCTCTGATGTCCTTCCATGCTGAGAGTTAGGGCCCTTGTCCAATCATGTTC
HC27 – 7: GAATTTTGCCCAAGTTTTTTCAGCTTTTCCCATTTTGTGTGTAATACGTACACACCGCAGGGTAATAACTG
HC27 – 8: GACGGGCTTCTTTCTAGACTGGCCAAAGACTTTGTCCACTTGTGCGCAGTTATCTATGCTGTCTCACCATAGAG
HC27 – 9: GGAGGTCTAGGCTGGAGCTGATCCAG
HC27 – 10: CCTCGTCCCCTGGTTGTTCTCTCAAGAAAAAGTATGCGTGATCATTTTGTC
HC27 – 11: AGAGAACAACCAGGGGACGAGG
HC27 – 12: GTCCACATAGTCCTCCAGAGAGAGAAG
HC27 – 13: GACCCAAGTCCAGTTCCCAACAACCAC
HC27 – 14: CCTCGTCCCCTGGTTGTCCTCTCAAGAGAGGAGGGAGTGTGAGCTTTTCC
HC27 – 15: AGAGGACAACCAGGGGACGAGGGGCTC
HC27 – 16: GCATGGGGAAGGGGCATTGTATGTAGG
HC27 – 17: CAGATCACACTGTCTGCTCACTTCAC
HC27 – 18: AAGGCAGCAGGATGGAAGCTGATGTCG
HC27 – 19: GCTGGAGGGCAACACAGGAAAGATGTGGGAATAAGTTTACTAGTCATACAGGCAGGAACCCCAGGCCCAGAGGTAGTGTCCCTGTGGGAGGGTCTCTTGCGCACACACCGCAGGGTAATAACTG
HC27 – 20: GATTTAAATGTCAATTGGTGAGTCTTCTGGGGCTTCCTACATACAATGCCCCTTCCCCATGCGCAGTTATCTATGCTGTCTCACCATAGAG

**BAC9, BAC (14+5) Method**

A self-replicating shuttle vector, termed pCAU, efficiently working in both *Saccharomyces cerevisiae* and *E. coli*, was constructed based on pBelo-CEN-URA published previously.^5^ In brief, *ARSH4* was amplified from *S. cerevisiae* genomic DNA using primers 878 and 879 (all primer sequences are listed below), with an *ApaL*I site followed by *AsiS*I and a *SexA*I introduced into either end. The fragment was digested with *ApaL*I and *SexA*I, and ligated with pBelo-CEN-URA digested with the same restriction enzymes to yield pCAU. This vector contains *S. cerevisiae* *CEN4*, *URA3* and *ARSH4* in the pBeloBAC11 backbone (New England BioLabs).

Three BACs derived from human chromosome 14 - CTD-2011A5 (BAC9), CTD-3148C6 (BAC14), CTD-2548B8 (BAC5) were purchased from Invitrogen/Thermo Fisher. The human genomic region encompassing IgHV3-74 to IgHV1-58 in BAC9 was isolated as a 185 kb *Not*I – fragment. BAC(14+5) was constructed from BAC14 and BAC5. The combined genomic regions in this BAC was isolated as a 210 kb *Bsi*wI - fragment including from 5’ to 3’: a 90.6 kb region derived from BAC14 containing 4.6 kb sequence overlapping with the 3’ of the *Not*I –fragment from BAC9 followed by a 86 kb region encompassing IgHV5-51 to IgHV1-45, a 1.7 kb synthetic region joining BAC14 and BAC5 with IgHV3-43 located in the centre, a 111.7 kb region derived from BAC5 encompassing IgHV3-21 to IgHV3-13, and a 6.1 kb region providing an overlap with the 5’ of Anabel (the BAC carrying human Ig constant regions).

BAC(14+5) was constructed in three steps all involving generating a circular YAC (cYAC) via homologous recombination in yeast and converting the cYAC to BAC as described previously. Firstly, a BAC vector - pCAU+GAP-BAC14,5, was generated by assembling the following 3 overlapping fragments in yeast: a 1.9 kb synthetic DNA (ordered from ThermoFisher) containing from 5’ to 3’: 116 bp sequence overlapping with the 5’ as well as 3’ end of the desired region in BAC14 with an unique *Rsr*II site in the centre, 1.6 kb IgHV3-43 gene [including 1.0 kb 5’ untranslated region (UTR) and 0.2 kb 3’ UTR], 106 bp sequence overlapping with the 5’ as well as 3’ end of the desired region in BAC5 with an unique *Pme*I site in the centre, and 38 bp sequence overlapping with the 5’ end of Anabel, a 6.1 kb PCR fragment corresponding to the 5’ of Anabel using primers 383 and 384, and an amplified pCAU vector using primers 1066 and 1088. Secondly, the pCAU+GAP-BAC14,5 vector was linearized with *Pme*I, and co-transformed with a 154 kb *Not*I – fragment isolated from BAC5 into yeast strain AB1380. The resulting BAC (~ 128 kb in length) had the desired region of BAC5 incorporated into the BAC vector via homologous recombination mediated by the homology ends to BAC5 exposed in the *Pme*I – linearized vector. Thirdly, the BAC carrying BAC5 from the second step was linearized with *Rsr*II to expose the homology ends to the desired region in BAC14, and co-transformed with a 114 kb *Sna*BI – fragment isolated from BAC14 to yield BAC(14+5).

Primers list:

| Primer 383 | CCTCCCATGATTCCAACACTG |
| --- | --- |
| Primer 384 | CTCACCGTCCACCACTGCTG |
| Primer 878 | GCGATCGCAAAGACGAAAGGGCCTCGTG |
| Primer 879 | ACCTGGTGATCGCCAACAAATACTACC |
| Primer 1066 | GCGGTGGGTCTCCCACGGGGGCAAACAGCAGTGGTGGACGGTGAGCGTACGGTTATCTATGCTGTCTCACCATAG |
| Primer 1088 | CTGTCAGCTGGAAGCAGTTAAGGTTGGCCTTTGTCTGTATTCGTACGCACACGCTTTTCAATTCAATTCATC |

DNA sequence synthesized by Thermo/Fisher:

CGTACGAATACAGACAAAGGCCAACCTTAACTGCTTCCAGCTGACAGGGGATGCTGTTTCGGACCGTCTGCAAGCTGAGGAGCAAGGAAGCCAGTCCAAGTCCCAAAACCTCGAAAGTCAGGTGGAAAAGTCGTTTATTGGGATAGTACCCACTACTACAATCAGTAAATGTTGGATACGATCAACAGCATGGTTCAATTCACAAGTACATATGATGAGTATAGTGAGCCAAAGCACATACATATGATTCCGTTTTATAAACTGTACAAAGTGAATACTCATTGGAAATTACATAACAAAGATCACTAACTGACTTCTCCATAGTAAGAGAAGCGAAGGTATAGGAGGGAGAAATTGTGAGAGACAAAGAGAAAATTGAGAGGCGAATTGATTTGTTTTCTCTGTGAATGGTCATTAGGTCAATGTTTGTCAAATGGTGAACATATTGTGTGAAGATTATATGTCTGTATTACTTCATTAAAGCTATTATAAATAAAAGTCTAATGTGGTAGAAAAAGATGAAGAGAGAAATAAAAATAATACAAGAAAAGTCATGAACTCCTGAATGAATTAACCCTTAGTTTTTCTCTATTACTTATAAAAACACCAAGATACAGCCAAATAATATCACGATATCATTATAAGAAGAGTGTTTTGTAAACCTCACTGGGAATTTATAGCTCTTTCCTAGAGTTAATTTTGGGAACAGTTGGATCCAATTGTGAGAAATGCAGGCTGGACACTGAGACTGGCTCTTATGAGATGTGAGCTCTTGTCTATGTCACATGGTCCTTCCATACTTGGGGGTTTACATTCACATCTGTAAATGAAGGAAACATTGACTCTCAAAGAACATATTTCATGTGCATGTAAAAGTATGAATGCTAGTGAGAATTAATTACTTATGAAGTATAATCACCCACATCCACTCTTGGACACAGCCCACTCTGAGGCATCTGTTACAGAACTCATTATATAGTAGGAGACATGCAAATAGGGTCCTCCCTCTGCTGATGAAAACCAGCCCAGCCCTGACCCTGCAGCTCTGGGAGAGGAGCCCCAGCCCTGAGATTCCCAGGTGTTTCCATTCGGTGATCAGCACTGAACACAGAGAACGCACCATGGAGTTTGGACTGAGCTGGGTTTTCCTTGTTGCTATTTTAAAAGGTGATTCATGGATAAATAGAGATGTTGAGTGTGAGTGAACATGAGTGAGAGAAACAGTGGATATGTGTGGCAGTGTCTGACCAGGGTGTCTCTGTGTTTGCAGGTGTCCAGTGTGAAGTGCAGCTGGTGGAGTCTGGGGGAGTCGTGGTACAGCCTGGGGGGTCCCTGAGACTCTCCTGTGCAGCCTCTGGATTCACCTTTGATGATTATACCATGCACTGGGTCCGTCAAGCTCCGGGGAAGGGTCTGGAGTGGGTCTCTCTTATTAGTTGGGATGGTGGTAGCACATACTATGCAGACTCTGTGAAGGGCCGATTCACCATCTCCAGAGACAACAGCAAAAACTCCCTGTATCTGCAAATGAACAGTCTGAGAACTGAGGACACCGCCTTGTATTACTGTGCAAAAGATACACAGTGAGGGGAAGTCAGCGAGAGCCCAGACAAAAACCTCGCTGCAGGAAGACAGGAGGGGCCTGGGCTGCAGAGGCCACTCAAGACACACTGAGCATAGGGTTAACTCTGGGACAAGTTGCTCAGGAAGGTTAAGAGCTGGTTTCCTTTCAGAGTCTTCACAATTTCTCCATCTAACAGTTTCCCCAGGAACCCTGTCGCAATGTCCTGCAGCACCTCAGACCACCTGTCCTGGTTCTATCAAGGAAGTTTAAACAATTTATGCAACCCACCTCATTTTCACTCATAAACCTATGGATTGACATCGCCTCCCATGATTCCAACACTGAGTTCAGACTTGTCACGTG
